# Independent contribution of polygenic risk for schizophrenia and cannabis use in predicting psychotic-like experiences in young adulthood: testing gene × environment moderation and mediation

**DOI:** 10.1017/S0033291721003378

**Published:** 2023-04

**Authors:** Laurent Elkrief, Bochao Lin, Mattia Marchi, Mohammad H Afzali, Tobias Banaschewski, Arun L. W. Bokde, Erin Burke Quinlan, Sylvane Desrivières, Herta Flor, Hugh Garavan, Penny Gowland, Andreas Heinz, Bernd Ittermann, Jean-Luc Martinot, Marie-Laure Paillère Martinot, Frauke Nees, Dimitri Papadopoulos Orfanos, Tomáš Paus, Luise Poustka, Sarah Hohmann, Juliane H. Fröhner, Michael N. Smolka, Henrik Walter, Robert Whelan, Gunter Schumann, Jurjen Luykx, Marco P. Boks, Patricia J. Conrod, the IMAGEN consortium

**Affiliations:** 1 Sainte-Justine Hospital Research Center, Montréal, Québec, Canada; 2 Département de psychiatrie et d'addictologie, Université de Montréal, Montréal, QC, Canada; 3 Department of Translational Neuroscience, Brain Center University Medical Center, Utrecht University, Utrecht, the Netherlands; 4 Department Psychiatry, Brain Center University Medical Center Utrecht, Utrecht, the Netherlands; 5 Department of Biomedical, Metabolic and Neural Sciences, University of Modena and Reggio Emilia, Via Giuseppe Campi, 287–41125 Modena, Italy; 6 Department of Child and Adolescent Psychiatry and Psychotherapy, Central Institute of Mental Health, Medical Faculty Mannheim, Heidelberg University, Square J5, 68159 Mannheim, Germany; 7 Discipline of Psychiatry, School of Medicine and Trinity College Institute of Neuroscience, Trinity College Dublin, Dublin 2, Ireland; 8 Centre for Population Neuroscience and Precision Medicine (PONS), Institute of Psychiatry, Psychology & Neuroscience, SGDP Centre, King's College London, United Kingdom; 9 Department of Cognitive and Clinical Neuroscience, Central Institute of Mental Health, Medical Faculty Mannheim, Heidelberg University, Square J5, Mannheim, Germany; 10 Department of Psychology, School of Social Sciences, University of Mannheim, 68131 Mannheim, Germany; 11 Departments of Psychiatry and Psychology, University of Vermont, 05405 Burlington, Vermont, USA; 12 Sir Peter Mansfield Imaging Centre School of Physics and Astronomy, University of Nottingham, University Park, Nottingham, United Kingdom; 13 Charité – Universitätsmedizin Berlin, Department of Psychiatry and Psychotherapy, Campus Charité Mitte, Charitéplatz 1, Berlin, Germany; 14 Physikalisch-Technische Bundesanstalt (PTB), Abbestr. 2 - 12, Berlin, Germany; 15 Institut National de la Santé et de la Recherche Médicale, INSERM Unit 1000 “Neuroimaging & Psychiatry”, University Paris Saclay, University Paris Descartes - Sorbonne Paris Cité; and Maison de Solenn, Paris, France; 16 Institut National de la Santé et de la Recherche Médicale, INSERM Unit 1000 “Neuroimaging & Psychiatry”, University Paris Sud, University Paris Descartes; and AP-HP.Sorbonne Université, Department of Child and Adolescent Psychiatry, Pitié-Salpêtrière Hospital, Paris, France; 17 NeuroSpin, CEA, Université Paris-Saclay, F-91191 Gif-sur-Yvette, France; 18 Bloorview Research Institute, Holland Bloorview Kids Rehabilitation Hospital, Toronto, Ontario, Canada; 19 Department of Child and Adolescent Psychiatry and Psychotherapy, University Medical Centre Göttingen, von-Siebold-Str. 5, 37075, Göttingen, Germany; 20 Department of Psychiatry and Neuroimaging Center, Technische Universität Dresden, Dresden, Germany; 21 School of Psychology and Global Brain Health Institute, Trinity College Dublin, Ireland;; 22 PONS Research Group, Dept of Psychiatry and Psychotherapy, Campus Charite Mitte, Humboldt University, Berlin and Leibniz Institute for Neurobiology, Magdeburg, Germany, and Institute for Science and Technology of Brain-inspired Intelligence (ISTBI), Fudan University, Shanghai, P.R. China.

**Keywords:** Polygenic risk score, cannabis, psychotic-like experience, psychosis risk

## Abstract

**Background:**

It has not yet been determined if the commonly reported cannabis–psychosis association is limited to individuals with pre-existing genetic risk for psychotic disorders.

**Methods:**

We examined whether the relationship between polygenic risk score for schizophrenia (PRS-Sz) and psychotic-like experiences (PLEs), as measured by the Community Assessment of Psychic Experiences-42 (CAPE-42) questionnaire, is mediated or moderated by lifetime cannabis use at 16 years of age in 1740 of the individuals of the European IMAGEN cohort. Secondary analysis examined the relationships between lifetime cannabis use, PRS-Sz and the various sub-scales of the CAPE-42. Sensitivity analyses including covariates, including a PRS for cannabis use, were conducted and results were replicated using data from 1223 individuals in the Dutch Utrecht cannabis cohort.

**Results:**

PRS-Sz significantly predicted cannabis use (*p* = 0.027) and PLE (*p* = 0.004) in the IMAGEN cohort. In the full model, considering PRS-Sz and covariates, cannabis use was also significantly associated with PLE in IMAGEN (*p* = 0.007). Results remained consistent in the Utrecht cohort and through sensitivity analyses. Nevertheless, there was no evidence of a mediation or moderation effects.

**Conclusions:**

These results suggest that cannabis use remains a risk factor for PLEs, over and above genetic vulnerability for schizophrenia. This research does not support the notion that the cannabis–psychosis link is limited to individuals who are genetically predisposed to psychosis and suggests a need for research focusing on cannabis-related processes in psychosis that cannot be explained by genetic vulnerability.

## Introduction

Cannabis use is a well-studied risk factor for psychosis, schizophrenia spectrum disorders and psychopathology in general. Meta-analysis and systematic reviews have consistently shown that there is a higher incidence of psychotic outcomes among cannabis users (Moore et al., 2007; Semple, McIntosh, & Lawrie, 2005) and that this relationship is dose dependent (Marconi, Di Forti, Lewis, Murray, & Vassos, 2016). Using cannabis during adolescence further increases risk for psychosis (Kelley et al., 2016; Mustonen et al., 2018), earlier onset of psychotic symptoms (Galvez-Buccollini et al., 2012) and worsened prognosis (Manrique-Garcia et al., 2014). Although the epidemiological evidence, along with some experimental evidence (D'Souza et al., 2004), suggests a causal link between cannabis use and psychosis, the nature of this relationship remains the focus of fierce debate (Forti, Morgan, Selten, Lynskey, & Murray, 2019; Sommer & van den Brink, 2019). Generally, three different hypotheses are used to explain the mechanisms of the cannabis–schizophrenia association: (1) the relationship is fully causal, i.e. cannabis use causes schizophrenia, (2) the relationship may be partially confounded by shared genetic and environmental confounders and/or reverse causation and (3) this link is entirely non-causal (Gillespie & Kendler, 2021; Hiemstra et al., 2018).

Considering that part of the aetiology of cannabis use and psychosis can be explained through heritable processes (Cardno et al., 1999; Verweij et al., 2010), recent large scale genome-wide association studies (GWASs) have demonstrated that multiple single-nucleotide polymorphisms (SNPs) are associated with risk for schizophrenia (Pardiñas et al., 2018), and predict cannabis use behaviours (Johnson et al., 2020; Pasman et al., 2018). Researchers can summarise the genetic risk for a disease through polygenic risk score (PRS) calculations, derived from the summary statistics generated in these large-scale GWASs. Although most PRSs for psychiatric diseases can currently only account for a small portion of the variance of disease [approximately <10% (Murray et al., 2021)], PRS can inform about shared genetic aetiology among complex traits, and can also be used to estimate the genetic risk to a trait at the individual level (Choi, Mak, & O'Reilly, 2020). In view of the purported cannabis–psychosis link, researchers have examined the link between polygenic risk score for schizophrenia (PRS-Sz) and cannabis use. PRS-Sz has been consistently associated with varying levels of cannabis use across numerous cohorts (Carey et al., 2016; Guloksuz et al., 2019; Hartz et al., 2017; Hiemstra et al., 2018; Jones et al., 2020; Verweij et al., 2017). Consequently, some have concluded that the relationship between PRS-Sz and cannabis use represents a pathway from genetic risk for schizophrenia to cannabis use (Jones et al., 2020), or that sensitivity to exposure to cannabis use is moderated by PRS-Sz (Guloksuz et al., 2019). In contrast, one highly powered study reported that PRS-Sz was not associated with cannabis use disorder in healthy controls, or patients with psychiatric disorders other than schizophrenia (Hjorthøj et al., 2021). Furthermore, they report that the association between prior cannabis use disorder and later development for schizophrenia was not altered after adjustment for PRS-Sz and PRS of other psychiatric disorders (Hjorthøj et al., 2021), suggesting that the association between cannabis use and development of schizophrenia is not explained by common genetic vulnerability (Hjorthøj et al., 2021). Nevertheless, the results of most studies utilising PRS-Sz, along with experiments employing discordant relative designs (Giordano, Ohlsson, Sundquist, Sundquist, & Kendler, 2015) and studies using Mendelian randomisation (MR) techniques (Gage et al., 2017; Pasman et al., 2018) support the second hypothesis – mainly that the relationship between schizophrenia and cannabis use is confounded by shared genetic vulnerability and reverse causation.

The relationship between cannabis use and psychosis development is particularly interesting in the adolescent ‘clinical high-risk for psychosis’ (Fusar-Poli, 2017) population. These individuals are at a high risk for psychosis in the presence of sub-clinical psychotic symptoms, functional decline and/or genetic risk (Fusar-Poli, 2017). As such, research on the developmental origins of psychosis risk has focused on the emergence of psychotic-like experiences (PLEs) during the adolescent period and how cannabis might influence such trajectories.

PLEs are highly prevalent sub-clinical psychotic symptoms (Ronald et al., 2014), reported in up to 7% of individuals (Linscott and van Os, 2013). Similar to the current models of symptomatology in patients along the psychotic spectrum, these sub-clinical symptoms have been further subdivided into various dimensions, such as positive, negative and affective symptoms (van Os & Reininghaus, 2016). Although these sub-clinical experiences are transitory in about 80% of individuals, PLEs are persistent in 20% of individuals (van Os et al., 2017). Moreover, the presence of PLEs in community samples is associated with increased odds for any mental disorder [odds ratio (OR) 3.08, 95% confidence interval (95% CI) 2.26–4.21], and psychotic disorders (OR 3.96, 95% CI 2.03–7.73) (Healy et al., 2019).

Considering the close relationship of PLEs to psychotic disorders, many have tested the hypothesis that cannabis use also increases one's risk for PLEs (see Ragazzi, Shuhama, Menezes, & Del-Ben, 2018 for a systematic review). One study found that cannabis use is significantly associated with the positive PLEs (*β* = 0.061, *p* < 1 × 10^−4^), even after controlling for numerous confounding factors (van Gastel et al., 2012). Another study found that the relationship between PLEs and cannabis use is increased in the heaviest of cannabis consumers (Schubart et al., 2011); in those who spend >€25/week on cannabis (i.e. heaviest users), there was an increased odds for various domains of PLE such as negative symptoms (OR 3.4, 95% CI 2.9–4.1), positive symptoms (OR 3.0, 95% CI 2.4–3.6) and depressive symptoms (OR 2.8, 95% CI 2.3–3.3) (Schubart et al., 2011). Furthermore, cannabis use has also been shown to temporally precede PLE in adolescent cohorts (Bourque, Afzali, & Conrod, 2018), but PLEs in childhood do not predict cannabis use (Jones et al., 2018a). Overall, the study of PLE in cohorts of cannabis users may be an interesting avenue to understand the nature and potential directionality of the cannabis–psychosis relationship.

PRS-Sz are also related to PLE. Although initial studies reported no relationship between PRS-Sz and PLEs (Derks et al., 2012; Zammit et al., 2014), more recent studies – with greater power – have found that PRS-Sz is associated with PLEs (Jones et al., 2016, 2018b; Pain et al., 2018; Taylor et al., 2019). But, there remains contradictory evidence in this field. For example, some have reported that PRS-Sz is related to the negative and affective symptom domains (Jones et al., 2016; Jones et al., 2018b), but not positive symptoms (hallucinations, paranoia and thought disturbance), whereas others have reported an association between PRS-Sz and positive symptoms (Pain et al., 2018; Taylor et al., 2019).

Thus, although the relationship between polygenic risk for schizophrenia and cannabis use has been consistently described in the literature, and the link between cannabis use and psychotic-like symptoms is shown to be significant, the relationship between all three factors (polygenic risk, cannabis use and PLE) is not yet fully understood. Although other studies have attempted to find environmental factors that mediate the relationship between PRS-Sz and cannabis use (Jones et al., 2020), to our knowledge no study has examined if cannabis use mediates the relationship between PRS-Sz and PLEs. Thus, considering that PRS-Sz may be directly or indirectly linked to cannabis use, the current study aims to investigate whether or not the pathway from genetic vulnerability to psychosis symptoms, is at least partially mediated by an indirect pathway through cannabis use.

In addition to the mediation hypothesis, we also test a moderation hypothesis, in which cannabis use might exacerbate genetic vulnerability to schizophrenia, and in turn increase the frequency of PLE. Clarifying the moderating role of genetic vulnerability on the relationship between cannabis and psychosis would also help to inform decision making with regards to guidelines for recreational cannabis in which individuals with a certain risk profile could be advised accordingly, in addition to the existing literature. These two hypotheses will be contrasted against a null hypothesis, which postulates that despite any potential common genetic vulnerability to cannabis use and psychosis risk, the relationship between cannabis use and psychosis risk holds, and is independent of (or in addition to) a common genetic vulnerability (i.e. cannot be explained by common genetic vulnerability). To test all hypotheses, we use a developmentally informed approach that focuses on temporal precedence to confirm mediation between variables. The current study uses data from two independent European cohorts: we use data from the IMAGEN (Schumann et al., 2010) study, a longitudinal study of over 2000 European adolescents, as a discovery sample, and aim to replicate those results in an independent European sample, the Utrecht cannabis cohort (Schubart et al., 2011). The use of the IMAGEN cohort is ideal considering that it allows for a longitudinal view of cannabis use and PLE development, during the critical years of adolescence. Furthermore, this cohort is relatively well powered to detect mediation effects, as similarly sized cohorts have attempted to discern such effects using similar phenotypes (Jones et al., 2020). This is compared with the cross-sectional Utrecht cannabis cohort, which is a cohort that has been enriched for PLE and heavy cannabis use; heavy cannabis use being a particularly strong risk factor for development of psychosis and PLE.

## Methods

### Participants

#### IMAGEN sample

The IMAGEN study is a longitudinal imaging genetics study of over 2000 healthy adolescents, mostly of European descent. Detailed descriptions of this study, genotyping procedures and data collection have previously been published (Schumann et al., 2010). The current study uses data for the 2087 who contributed their genetic data. The multicentric IMAGEN project had obtained ethical approval by the local ethics committees (at their respective sites) and written informed consent from all participants and their legal guardians. The parents and adolescents provided written informed consent and assent, respectively at 14 and 16, and then participants gave full consent at 18 and 21 years of age.

#### Utrecht cannabis cohort

Data from the Utrecht cannabis cohort come from a subset (*N* = 1223) of a large (*N* = 17 698) cohort of young Dutch participants, for which genetic, cannabis use and PLE data were available. Detailed descriptions of recruitment methods, genotyping procedures and data collection were previously published (Boks et al., 2020; Schubart et al., 2011). Participants gave online informed consent, and the study received approval by the University Medical Centre Utrecht medical ethical commission. Of note is the enrichment for the extremes in PLE and cannabis use data in the Utrecht cannabis cohort. To increase power for gene × environment interactions in previous studies (Boks et al., 2007), data from individuals from the general population were combined with data of participants selected from the top or bottom quintile of total PLE scores, who are either non-users (<2 lifetime exposures to cannabis) or heavy users (i.e. current expenditure for personal cannabis use exceeded €10 weekly).

### Phenotype measures

#### Cannabis use measures

IMAGEN participants were repeatedly assessed for cannabis use at 14, 16, 18 and 21 years of age using questions taken from the European School Survey of Alcohol and other Drugs (ESPAD) questionnaire. The ESPAD is a self-report questionnaire that measures the use of various drugs of abuse, including cannabis (Hibell et al., 1997, 2004). With very few participants reporting cannabis use at 14 years of age, we focus our analyses on data that were collected at the 16-year-old assessment, using responses to the question ‘On how many occasions in your whole lifetime have you used marijuana (grass, pot) or hashish (hash, hash oil)?’. Answers are scored on a scale ranging from 0 to 6: ‘0’ = 0, ‘1–2 times’ = 1, ‘3–5 times’ = 2, ‘6–9 times’ = 3, ‘10–19 times’ = 4, ‘20–39 times’ = 5, ‘40 or more times’ = 6. In the Utrecht cannabis cohort, lifetime cannabis use data were reported according to the following categories: never = 0, ‘1 time’ = 1, ‘2 times’ = 2, ‘5–9 times’ = 3, ‘>10 times’ = 4.

#### PLE measures

PLE data for both cohorts were drawn from the Community Assessment of Psychic Experiences-42 (CAPE-42) questionnaire (Stefanis et al., 2002). CAPE-42 is a widely used self-report questionnaire that reliably measures lifetime PLEs (Mark & Toulopoulou, 2016). The CAPE-42 has three subscales that measure positive, negative and depressive symptom dimensions. The CAPE-42 measures frequency of symptoms, along with distress caused by symptoms. We only analyse frequency scores as distress and frequency scores are highly correlated in these cohorts (*r* > 0.80). In the primary analyses, we use the sum total of frequency scores, whereas we look at the various sub-dimensions in the secondary analysis. Due to the skewed distribution of scores, the log-transformed sum score of each individual dimension and total score of the frequency of symptoms was used. We used CAPE-42 data from the 18-year-old follow-up for the IMAGEN cohort.

### Genetic data

#### IMAGEN

The genotyping was conducted using the Illumina Quad 610 chip and 660Wq at the ‘Centre National de Genotypage’ (Paris, France). Non-imputed autosomal SNPs are used for this study (498 892 SNPs). Following all quality control steps, genetic data (468 170 SNPs) remained for 1740 individuals. Baseline quality control steps and principal component analysis to control for ancestry are described in online Supplementary materials.

#### Utrecht

The genotyping in this cohort was conducted using either the Illumina^®^ HumanOmniExpress (733 202 SNPs; 576 individuals) or the Illumina^®^ Human610-Quad Beadchip (620 901 SNPs; 768 individuals). The CannabisQuest cohort genetic dataset was also imputed, as described in Boks et al. (2020), using the HapMap III release 24 via Beagle 5.2 imputation server (Browning and Browning, 2009). As with the IMAGEN sample, quality control steps and principal component analysis for ancestry are described in online Supplementary materials. After all quality control steps, a total of 5 173 601 SNPs and 1126 individuals remained for analysis.

### Analysis

#### Polygenic risk scores

PRS-Sz were constructed for each of the IMAGEN and Utrecht cannabis individuals, who passed genetic quality control. PRS-Sz were built using data from the most recent schizophrenia GWASs based on 40 675 cases and 64 643 controls (Pardiñas et al., 2018) as a training set (for description of base set, see online Supplementary materials). PRSs were built using PRScs (Ge, Chen, Ni, Feng, & Smoller, 2019) and PLINK1.9 (Purcell). PRScs is used to infer posterior SNP effect sizes, by placing a continuous shrinkage prior on SNP effect sizes reported in the most recent schizophrenia GWASs (Ge et al., 2019), as well as an external LD reference panel. Here, we use the publically available 1000 Genomes Project phase 3 panel (https://github.com/getian107/PRScs). To calculate posterior effect sizes, in both cohorts, we use the default settings of PRScs, described in more detail in online Supplementary materials. After calculation of posterior effect sizes, PRSs were calculated using the ‘--score’ function and SUM modifier in PLINK1.9. After quality control, 321 567 variants are used to calculate PRS in the IMAGEN cohort, and 763 754 SNPs in the Utrecht cannabis cohort.

We aligned our analyses closely to the replication study, using the same protocol to create PRS, and, in both IMAGEN and Utrecht cannabis cohorts. To ease interpretability of results, we scale the PRS, using the scale function in R (R Core Team, 2020). Using the same methods, we created a PRS for cannabis use (PRS-Can), using publicly available data from the GWAS studying lifetime cannabis use (Pasman et al., 2018) (a detailed description of the base set can be found in online Supplementary materials), to be used as a potential confounder.

#### Statistical analysis

Multiple multinomial linear regressions were used to assess the relationships between PRS-Sz, cannabis use and PLEs in both cohorts. For our primary analyses, we examine four distinct models:

Model 1: The relationship between PRS-Sz, independent variable (IV), and total CAPE score (log-transformed), dependent variable (DV).

Model 2: The relationship between PRS-Sz (IV) and lifetime cannabis use (DV).

Model 3: The relationship between cannabis use (IV) and CAPE scores (DV), when accounting for PRS-SZ.

Model 4: The interaction between PRS-Sz and cannabis use as predictors of CAPE scores (moderation analysis).

We performed mediation analysis using maximum likelihood estimation (MLE) path analysis to assess the effect of PRS-Sz on PLE scores through the possible mediation effect of cannabis use. As alluded to above, our hypothesis is that cannabis use (M) mediates the relationship between PRS-Sz (independent variable; IV) and PLE (dependent variable; DV). We report the bias-corrected bootstrap 95% CI for the indirect effect, using an adjusted bootstrap percentile method (BCa), based on 5000 bootstrap samples. We use the MLE to handle missing data. All statistical analyses are performed in R (R Core Team, 2020). Mediation analysis was performed via the ‘lavaan’ (Rosseel et al., 2021) and ‘tidySEM’ packages (van Lissa, 2021). An *α* = 0.05 was set for significance. Mediation analysis is only executed using the IMAGEN data as this dataset is the only sample that assessed cannabis use some years before the PLE assessment and therefore the only dataset that can provide a true estimate of a longitudinal relationship.

The association between PRS-Sz, cannabis use and the various sub-domains of the CAPE-42 questionnaire was analysed in secondary analyses, through linear regression, in both cohorts. For all statistical analyses of the IMAGEN cohort, we consider the following potential confounders: sex, the first-six genetic principal components (PCs). In the Utrecht cannabis cohort, we add age as a potential confounder. Finally, as a sensitivity analysis we include PRS-Can as a potential confounder in regression analyses for the IMAGEN cohort.

## Results

### Sample characteristics

Characteristics of participants who passed genetic QC and responded to cannabis use and PLE questionnaire data are detailed in online Supplementary Table S2. Data from a total of 1740 individuals were used to calculate PRS-Sz in IMAGEN, and 1223 individuals in Utrecht cannabis cohort. In both IMAGEN and Utrecht cannabis cohort samples, males report higher cannabis use compared to females (*p* < 0.001). The total frequency of CAPE-42 symptoms reported is significantly greater in males (*p* < 0.001) in the IMAGEN cohort. There was no difference in the reported total CAPE-42 symptoms between males and females in the Utrecht cannabis cohort (online Supplementary Table S2). Female participants in both cohorts report significantly higher scores in the depression symptom sub-scale of the CAPE-42 (*p* < 0.001). Finally, the mean age of the Utrecht cannabis cohort is 20.5 years.

### Regression models

#### Model 1: association of PRS-Sz with PLE

PRS-Sz predicted PLE in both cohorts (Table 1), when accounting for covariates. PRS-Sz was significantly associated with CAPE-42 scores in both cohorts (*β*_IMAGEN_ = 0.015 *p* = 0.004, *R*^2^ = 0.019; *β*_Utrecht cannabis_ = 0.021, *p* = 0.0003, *R*^2^ = 0.016). The results for the full regression model, including covariates, are shown in online Supplementary Table S3.

**Table 1.** Linear regression models

	Independent variable	*β*	Std. error	*p*	*R*^2^
*IMAGEN*					
Model 1					*0*.*019*
	*PRS-Sz*	0.015	0.005	**4.43 × 10^−3^**	
Model 2					0.041
	PRS-Sz	0.097	0.044	**2.72 × 10^−2^**	
Model 3					0.028
	PRS-Sz	0.014	0.005	**1.03 × 10^−2^**	
	Cannabis use	0.009	0.003	**6.91 × 10^−3^**	
Model 4					0.028
	PRS-Sz	0.016	0.006	**1.09 × 10^−2^**	
	Cannabis use	0.009	0.003	**6.29 × 10^−3^**	
	PRS:cannabis	−0.002	0.004	5.78 × 10^−1^	
*Utrecht*					
Model 1					0.016
	*PRS-Sz*	0.021	0.006	**3.11 × 10^−4^**	
Model 2					0.17
	PRS-Sz	0.235	0.049	**2.21 × 10^−6^**	
Model 3					0.037
	PRS-Sz	0.017	0.006	**3.48 × 10^−3^**	
	Cannabis use	0.017	0.003	**3.61 × 10^−7^**	
Model 4					0.037
	PRS-Sz	0.017	0.008	**4.13 × 10^−2^**	
	Cannabis use	0.017	0.003	**3.66 × 10^−7^**	
	PRS:cannabis	−0.00004	0.003	9.88 × 10^−1^	

*β*, main effect size; std. error, standard error. Bold values indicate *p* < 0.05.

This table shows results of the effects of the independent variables in linear regression models. Dependent variable is log-transformed CAPE-42 scores for models 1-3-4, whereas dependent variable for model 2 is lifetime cannabis use. We considered the first six PC and sex as covariates for all analyses and age is included for all analyses of the Utrecht cannabis cohort.

#### Model 2: association of PRS-Sz with cannabis use

After accounting for covariates, the PRS-Sz predicted cannabis use in both cohorts (*β*_IMAGEN_ = 0.097, *p* = 0.027, *R*^2^ = 0.041; *β*_Utrecht cannabis_ = 0.24, *p* < 0.00001, *R*^2^ = 0.17, Table 1). The results for the regression model, including covariates, are shown in online Supplementary Table S4.

#### Model 3: association of cannabis use with PLE

Cannabis use significantly predicted PLE in both cohorts (*β*_IMAGEN_ = 0.0091, *p* = 0.007, *R*^2^ = 0.028; *β*_Utrecht cannabis_ = 0.017, *p* < 0.00001, *R*^2^ = 0.037; Table 1), when considering PRS-Sz and all confounders. Moreover, PRS-Sz remained as a significant predictor (*p* < 0.05) within this model in both cohorts (online Supplementary Table S5).

#### Model 4: moderation analysis

In our moderation model, the interaction between cannabis use and PRS-Sz was also not significant (*p* > 0.05; online Supplementary Table S6), suggesting that both cannabis use and PRS-Sz independently predict PLE.

#### Mediation analysis

**Fig. 1.**
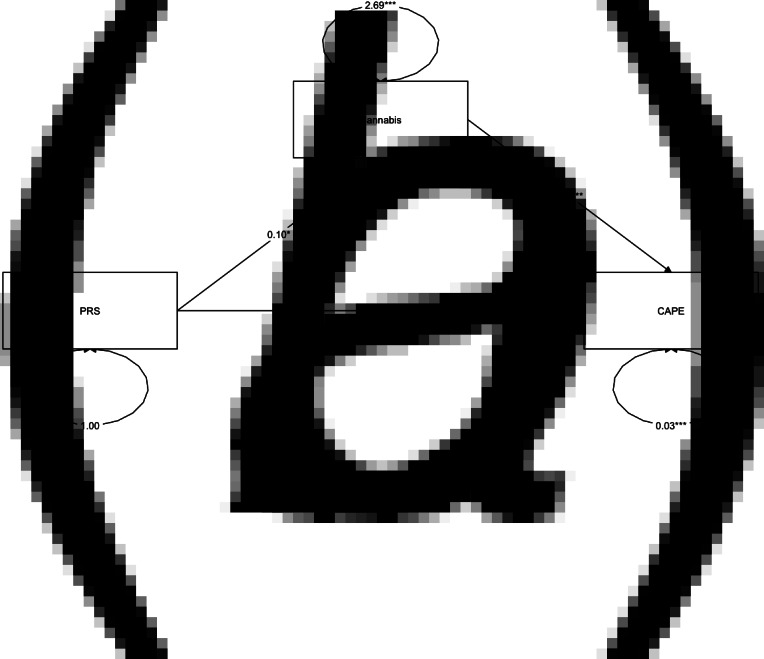
Results of mediation analysis. Independent variable = polygenic risk score for schizophrenia (PRS-Sz), dependent variable = log Total of CAPE-42 (CAPE), Mediator = cannabis use. Although the effects of each path (a, b and c) are significant, the indirect path of PRS-Sz on PLEs as measured by the CAPE-42 questionnaire through cannabis use was not significant (*p* > 0.05). The total effect was significant. The estimate is shown between arrows, with * signifying statistical significance. *p < 0.05, **p < 0.01.

Although PRS-Sz predicted PLE at 18 years of age (Fig. 1, path c), and lifetime cannabis use at 16 years of age (Fig. 1, path a), and that cannabis use (16 years) was significantly associated with PLE (18 years) (Fig. 1, path b), there was no evidence that PRS-Sz influences PLE through previous cannabis use (*β* = 8.79 × 10^−4^, 95% CI −1.23 × 10^−4^ to 1.88 × 10^−3^, *p* = 0.08; Table 2). For full results of path analysis, including covariates, see online Supplementary Table S7. Taken together, these results suggest that both cannabis use and PRS-Sz independently predict PLE.

**Table 2.** Mediation path analysis

Dependent variable	Independent variable	Label	*β*	s.e.	*z*	*p* value	CI, lower	CI, upper
CAPE								
	PRS	c	0.014	0.005	2.698	**0.007**	0.004	0.024
	Cannabis	b	0.009	0.003	2.721	**0.007**	0.003	0.016
Cannabis								
	PRS	a	0.097	0.044	2.217	**0.027**	0.011	0.182
Pathway effects								
Cannabis indirect effect		a × b	0.001	0.001	1.719	0.086	0.000	0.002
Total effect		c + (a × b)	0.015	0.005	2.865	**0.004**	0.005	0.025

*β*, main effect size; s.e., standard error. Bold values indicate *p* < 0.05.

We report the results of the path analysis examining the link between PRS-Sz and PLE, and whether there is a significant indirect effect (mediation) through cannabis use. Here, we report, estimates (*β*), s.e., *z* statistics and *p* value. We considered the first six PC and sex as covariates for all analyses. CIs are calculated adjusted bootstrap percentile method (BCa) based on 5000 bootstrap samples.

#### Secondary analysis

The relationship PRS-Sz, cannabis use and the different sub-domains in the CAPE-42 questionnaire was studied. In the IMAGEN cohort (Table 3; online Supplementary Table S8), PRS-Sz was significantly associated with the depression subscale (*β*_IMAGEN_ = 0.018, *p* = 0.01) and positive subscale (*β*_IMAGEN_ = 0.011, *p* = 0.02). Moreover, cannabis use was predictive of the depressive subscale (*β*_IMAGEN_ = 0.01, *p* = 0.02) and negative symptoms subscale (*β*_IMAGEN_ = 0.013, *p* = 0.003). Path analysis was also performed, for the IMAGEN cohort, to detect mediation effects of cannabis onto the different subscales, but it was not significant (*p* < 0.05; online Supplementary Table S10). On the contrary, in the Utrecht cannabis cohort, cannabis use was significantly associated with all three sub-domains (*p* < 0.005, Table 3, online Supplementary Table S9), whereas PRS-Sz was significantly associated with the depressive and negative subscales (*p* < 0.005; online Supplementary Table S9)

**Table 3.** Predictive value of PRS-Sz and cannabis use on CAPE-42 subscales

	Independent variable	*β*	Std. error	*p*	*R*^2^
*IMAGEN*					
Depressive sub-scale					0.065
	PRS-Sz	0.018	0.007	**1.12 × 10^−2^**	
	Cannabis use	0.010	0.004	**2.47 × 10^−2^**	
Negative sub-scale					0.020
	PRS-Sz	0.014	0.007	5.28 × 10^−2^	
	Cannabis use	0.013	0.005	**3.84 × 10^−3^**	
Positive sub-scale					0.021
	PRS-Sz	0.011	0.005	**2.76 × 10^−2^**	
	Cannabis use	0.004	0.003	1.68 × 10^−1^	
*Utrecht*					
Depressive sub-scale					0.065
	PRS-Sz	0.022	0.007	**1.10 × 10^−3^**	
	Cannabis use	0.010	0.004	**7.17 × 10^−3^**	
Negative sub-scale					0.033
	PRS-Sz	0.023	0.007	**1.85 × 10^−3^**	
	Cannabis use	0.019	0.004	**7.17 × 10^−6^**	
Positive sub-scale					0.039
	PRS-Sz	0.011	0.006	5.88 × 10^−2^	
	Cannabis use	0.017	0.003	**9.24 × 10^−8^**	

*β*, main effect size; std. error, standard error. Bold values indicate *p* < 0.05.

This table shows results of the effects of the independent variables in linear regression models on the various sub-scales of the CAPE-42. Dependent variable is log-transformed CAPE-42 scores for each subscale. We considered the first six PC and sex as covariates for all analyses and age is included for all analyses of the Utrecht cannabis cohort.

#### Sensitivity analysis

In the IMAGEN cohort, the three significant linear models (models 1–3) were reassessed considering PRS-Can as a potential confounder. The lifetime cannabis use PRS (PRS-Can) did not predict cannabis use measures at 16 years of age, or CAPE scores at 18 years of age (*p* > 0.5). After including PRS-Cannabis a covariable in model 3, both PRS-Sz and cannabis use remained significant (*β*_PRS-Sz_ = 0.014, *p* = 0.01, *β*_Cannabis Use_ = 0.009, *p* = 0.008). Moreover, PRS-Sz significantly predicted cannabis use (model 2), after inclusion of PRS-Can into the regression (*β* = 0.095, *p* = 0.03, online Supplementary Table S11).

## Discussion

In this study, we examine whether polygenic risk for schizophrenia predicts cannabis use, and higher levels of PLEs, in two independent European ancestry cohorts. Furthermore, we explore potential hypotheses through mediation and moderation analyses. Our results demonstrate that cannabis use can be reliably predicted by PRS-Sz, strengthening the existing literature (Carey et al., 2016; Hartz et al., 2017; Hiemstra et al., 2018; Jones et al., 2020). The evidence of association between PRS-Sz and cannabis use in the IMAGEN cohort has previously demonstrated by French et al., that cannabis use at 14 years of age interacted with PRS-Sz in decreasing cortical thickness from 14.5 to 18.5 years old (French et al., 2015). Here, we extend these findings by showing that the PRS-Sz predicts PLE. This too is in line with other study, using a variety in PLE assessments in various sub-domains (Jones et al., 2016; Jones et al., 2018b; Pain et al., 2018; Taylor et al., 2019). The current study confirms that PRS-Sz and cannabis use are linked to risk for PLEs overall and in the depressive and domains in both samples.

Considering the abundance of observational evidence showing temporal precedence of cannabis use in risk for psychosis, a reasonable alternative to a causal hypothesis is the proposal that cannabis use and PLE are explained through common genetic risk. However, our findings do not confirm this explanation, despite showing that PRS-Sz is correlated with both PLE and cannabis outcomes in both cohorts. This is in line with recent study that reported that various classes of cannabis use were associated with increased risk for psychotic experiences, even after adjusting for family history of schizophrenia (Jones et al., 2018a) and other study adjusting for PRS-Sz (Jones et al., 2020). To our knowledge, this is the first study to examine if cannabis use mediates the relationship between PRS-Sz and PLE. Through our longitudinal design, our analyses did not find any evidence to support mediation nor moderation hypotheses that explain the relationship between lifetime cannabis use and PLE. Consequently, these null findings suggest that despite the common genetic vulnerability of psychotic experiences, cannabis use and schizophrenia (Barkhuizen, Pain, Dudbridge, & Ronald, 2020; Pasman et al., 2018), both PRS-Sz and cannabis use independently increase one's risk for PLE, leaving room for alternative explanations of the cannabis–psychosis relationship.

Two recent studies employed MR technique to investigate causal links between cannabis use and schizophrenia (Gage et al., 2017; Vaucher et al., 2018). In both of these studies, there was weak evidence to support the causal hypothesis in the direction schizophrenia to cannabis use, while the reverse relationship was strong (Gage et al., 2017; Vaucher et al., 2018). Although these studies are limited by the power of the respective GWASs used, recent study has called into question causal inferences made in MR studies of complex traits (O'Connor & Price, 2018), and suggest the use of a latent causal variable (LCV) instead. In LCV models, genetic correlation between ‘two traits is mediated by a latent variable which has a causal effect on each trait’ (O'Connor & Price, 2018). Accordingly, a recent study examined the causal link between schizophrenia and lifetime cannabis use employing LCV and found no evidence for a causal genetic link between the two (Jang et al., 2020). Taken together, these reports do not preclude the possibility of a causal mechanism linking cannabis use to psychosis. Instead, they – along with the results presented above – suggest that psychosis or psychosis risk, and cannabis use may be linked through another environmental mediator rather than being linked through a common genetic predisposition.

The findings of the current study suggest that the variance in cannabis use that is most linked to PLEs is that which is not accounted for by PRS-Sz. This is interesting and suggests that future studies could focus on environmental factors influencing cannabis behaviours, such as the type of cannabis used, or available in a given population, the effects of advertisements endorsed by the cannabis industry, differing legalisation frameworks, and cannabis potency, when attempting to understand the link between cannabis and psychosis.

In secondary analysis of the current study, PRS-Sz was associated with depressive and positive sub-domains of the CAPE-42 in the IMAGEN cohort, whereas in the older Utrecht cannabis cohort, PRS-Sz was associated with depressive and negative sub-domains, with results trending towards significance for the positive sub-domain (*p* = 0.058). Some previous reports found no association between PLE-Sz and positive symptoms in adolescent populations (Jones et al., 2016, 2018b; Zammit et al., 2014). Nevertheless, one study has reported an association between PRS-Sz and positive PLE symptoms in their adolescent cohort (Pain et al., 2018), however, only when considering non-zero responders, i.e. those who have already manifested positive symptoms. Some have argued that the previously reported associations between PLE-Sz and the positive symptom domain in young adult populations can be explained by the fact that the genetic overlap between positive and negative psychotic experiences and schizophrenia might be stronger in adulthood than in adolescence (Barkhuizen et al., 2020). As previously suggested by Jones et al. (2016), this would imply that genetic risk for schizophrenia is in fact associated with positive PLE, but that this risk may be expressed in young adulthood rather than adolescence. Our results seem to be in line with this interpretation, considering that PLE in the IMAGEN cohort are reported at 18, i.e. the beginning of young adulthood. On the contrary, other environmental risk factors – such as cannabis use – may be what cause these same positive symptoms in adolescents.

### Limitations

Although the findings of this study are consistent with previous independent studies, this study is not without limitations and results should be interpreted accordingly. First, PRSs can only explain small portions of the variances of the phenotypes they study (Murray et al., 2021). In this study, PRS-Sz explained up to 17% of the variance of cannabis use and 1.9% of variance of PLE, when accounting for confounders. PRS also only incorporates data from common genetic variants; as such a significant portion of the genetic effects may not be captured through the PRS, such as the effects of rare variants and copy number variants, which also may play a role in the pathogenesis of schizophrenia (Malhotra & Sebat, 2012). Although a thorough imputation procedure was implemented to deal with missing data (Karahalios, Baglietto, Carlin, English, & Simpson, 2012), the IMAGEN dataset had several missing data points, which could bias our results. Next, considering the self-report nature of our phenotypic measures, our results may be at risk for measurement error, due to underreporting of symptoms, leading to weakened power. Moreover, we use the PRS-Sz – which was built to predict outcomes of clinical schizophrenia in adults – to predict PLEs in adolescent and young adult populations. Although the PRS-Sz has been used to reliably predict PLEs (Jones et al., 2016, 2018b; Pain et al., 2018; Taylor et al., 2019), the most discriminant SNPs for PLE may have not been captured by our PRS. However, considering the genetic overlap between schizophrenia and PLE (Barkhuizen et al., 2020), our significant result remains informative. Although our results consistently show that PRS-Sz is associated with lifetime cannabis use, we cannot make any conclusions about the effects of recent or current cannabis use. This is an important limitation considering the recent literature which demonstrates that current cannabis use is significantly associated with psychotic experiences in the general population, but that lifetime cannabis use is not (Quattrone et al., 2020). Moreover, our lifetime cannabis measure cannot account for potency or dose effects. This is an important consideration, as previous meta-analysis has shown heavy cannabis use with high tetrahydrocannabinol (THC) content poses a particular risk for psychosis (Marconi et al., 2016). Thus, future studies could consider current use, and dose–response, when examining mediating and moderating effects of cannabis on the PRS-Sz–psychotic experience association.

## Conclusion

In conclusion, although the current study could not confirm a mediated pathway between schizophrenia risk and PLE through cannabis use, the results contribute to the literature by showing the positive relationship between cannabis and future psychotic-like symptoms, while controlling for genetic vulnerability. Although we do not confirm any causal hypotheses, this result is important because, while cannabis producers would like to claim that cannabis use is only contra-indicated for individuals with a personal or family history of psychosis, the current findings suggest that cannabis use remains a risk factor for PLEs, over and above known genetic vulnerability for schizophrenia. Moreover, there was no evidence that genetically vulnerable individuals were more susceptible to the psychosis-related outcomes of adolescent onset cannabis use. As suggested by other authors (Jones et al., 2016), identifying a causal mechanism in the pathway from cannabis use to psychosis is extremely important for the development of targeted preventative interventions aimed at reducing cannabis use and/or schizophrenia risk.

## Supplementary material

For supplementary material accompanying this paper visit https://doi.org/10.1017/S0033291721003378. click here to view supplementary material
