# Supplementary material for: Independent contribution of polygenic risk for schizophrenia and cannabis use in predicting psychotic-like experiences in young adulthood: testing gene × environment moderation and mediation
Source: Psychol Med. 2021 Sep 23;53(5):1759–69. doi: 10.1017/S0033291721003378 (PMC10106286; doi:10.1017/S0033291721003378)
Supplement: Supplementary file 1 [file S0033291721003378sup.zip › S0033291721003378sup002.docx]

**Independent contribution of polygenic risk for schizophrenia and cannabis use in predicting psychotic-like experiences in young adulthood: Testing gene x environment moderation and mediation.**

Laurent Elkrief ^1*^, Bochao Lin^2^, Mattia Marchi^3,4^, Mohammad H Afzali^1,5^, Tobias Banaschewski^6^; Arun L.W. Bokde^7^, Erin Burke Quinlan^8^, Sylvane Desrivières^8^, Herta Flor^9,10^, Hugh Garavan^11^, Penny Gowland ^12^, Andreas Heinz^13^, Bernd Ittermann^14^, Jean-Luc Martinot ^15^, Marie-Laure Paillère Martinot ^16^, Frauke Nees ^6, 9^, Dimitri Papadopoulos Orfanos^11^, Tomáš Paus^17^, Luise Poustka^1^^8^, Sarah Hohmann^6^, Juliane H. Fröhner^19^, Michael N. Smolka^19^, Henrik Walter^13^, Robert Whelan^20^, Gunter Schumann^8, 22^, Jurjen Luykx ^2,23,24^, Marco P. Boks^3^, Patricia J. Conrod ^1,5^, and the IMAGEN consortium.

*Corresponding Author

**Author Affiliations:**

1. Sainte-Justine Hospital Research Center, Montréal, Québec, Canada.
2. Department of Translational Neuroscience, Brain Center University Medical Center, Utrecht University, Utrecht, the Netherlands

3. Department Psychiatry, Brain Center University Medical Center Utrecht, Utrecht, the Netherlands

4. Department of Biomedical, Metabolic and Neural Sciences, University of Modena and Reggio Emilia, Via Giuseppe Campi, 287 – 41125 Modena, Italy.

5. Département de psychiatrie et d’addictologie, Université de Montréal, Montréal, QC, Canada

6. Department of Child and Adolescent Psychiatry and Psychotherapy, Central Institute of Mental Health, Medical Faculty Mannheim, Heidelberg University, Square J5, 68159 Mannheim, Germany;

7. Discipline of Psychiatry, School of Medicine and Trinity College Institute of Neuroscience, Trinity College Dublin, Dublin 2, Ireland;

8. Centre for Population Neuroscience and Precision Medicine (PONS), Institute of Psychiatry, Psychology & Neuroscience, SGDP Centre, King’s College London, United Kingdom;

9. Department of Cognitive and Clinical Neuroscience, Central Institute of Mental Health, Medical Faculty Mannheim, Heidelberg University, Square J5, Mannheim, Germany;

10. Department of Psychology, School of Social Sciences, University of Mannheim, 68131 Mannheim, Germany;

11. Departments of Psychiatry and Psychology, University of Vermont, 05405 Burlington, Vermont, USA;

12. Sir Peter Mansfield Imaging Centre School of Physics and Astronomy, University of Nottingham, University Park, Nottingham, United Kingdom;

13. Charité – Universitätsmedizin Berlin, Department of Psychiatry and Psychotherapy, Campus Charité Mitte, Charitéplatz 1, Berlin, Germany;

14. Physikalisch-Technische Bundesanstalt (PTB), Abbestr. 2 - 12, Berlin, Germany;

15. Institut National de la Santé et de la Recherche Médicale, INSERM Unit 1000 “Neuroimaging & Psychiatry”, University Paris Saclay, University Paris Descartes - Sorbonne Paris Cité; and Maison de Solenn, Paris, France;

16. Institut National de la Santé et de la Recherche Médicale, INSERM Unit 1000 “Neuroimaging & Psychiatry”, University Paris Sud, University Paris Descartes; and AP-HP.Sorbonne Université, Department of Child and Adolescent Psychiatry, Pitié-Salpêtrière Hospital, Paris, France;

17. NeuroSpin, CEA, Université Paris-Saclay, F-91191 Gif-sur-Yvette, France

18. Bloorview Research Institute, Holland Bloorview Kids Rehabilitation Hospital, Toronto, Ontario, Canada.

19. Department of Child and Adolescent Psychiatry and Psychotherapy, University Medical Centre Göttingen, von-Siebold-Str. 5, 37075, Göttingen, Germany;

20. Department of Psychiatry and Neuroimaging Center, Technische Universität Dresden, Dresden, Germany;

21. School of Psychology and Global Brain Health Institute, Trinity College Dublin, Ireland;

22. PONS Research Group, Dept of Psychiatry and Psychotherapy, Campus Charite Mitte, Humboldt University, Berlin and Leibniz Institute for Neurobiology, Magdeburg, Germany, and Institute for Science and Technology of Brain-inspired Intelligence (ISTBI), Fudan University, Shanghai, P.R. China.

23. Department of Psychiatry, UMC Utrecht Brain Center, University Medical Center Utrecht, Utrecht

University, Utrecht, The Netherlands.

24. Outpatient second opinion clinic, GGNet Mental Health, Warnsveld, The Netherlands.

Corresponding author contact information:

Laurent Elkrief, CHU Ste Justine, Centre de Recherche, 3175 Ch Cote Ste-Catherine, Montreal, QC H3T1C5, [laurent.elkrief@umontreal.ca](mailto:laurent.elkrief@umontreal.ca)

Running Head: **Predictors of psychotic-like experiences**

**Supplemental material**

**Imagen Dataset:**

*IMAGEN Sample*

The IMAGEN study is a longitudinal imaging genetics study of 2087 healthy adolescents, mostly of European descent. Detailed descriptions of this study, genotyping procedures, and data collection have previously been published[1]. The current study uses data for all 2087 individuals who completed the IMAGEN assessment battery at 16, and 18 and who contributed their genetic data at 14 years of age. The multicentric IMAGEN project had obtained ethical approval by the local ethics committees (at their respective sites) and written informed consent from all participants and their legal guardians. The data to be used in this study are genetic, biological sex, behavioural measures (cannabis use), and psychotic-like experiences measures (CAPE questionnaire). The parents and adolescents provided written informed consent and assent, respectively at 14 and 16, and then participants gave full consent at 18 and 21 years of age. IMAGEN participants at 14, 16, 18 and 21 years of age were repeatedly assessed on cannabis use outcomes using the ESPAD questionnaire, and on psychotic like experiences using the CAPE questionnaire at 18 and 21 years of age. These questionnaires are completed by participants at home via the Psytools portal. Data is then de-identified through a two-step procedure were participants genetic and neuroimaging data are linked to the cognitive psytools data using two “pseudocodes” to ensure personal and test center anonymity [1].

*CannabisQuest Cohort*

This study recruited over 20,000 Dutch-speaking adolescents and young adults. Recruitment strategies are described in more detail in [2]. Participants answered questions on cannabis use history, along with the Community Assessment of Psychic Experiences questionnaire and provided their age, educational level and contact details. Data was collected from June 2006 to February 2009, which resulted in 21 838 participants. The assessment included two verification questions to protect against random answers, if participants failed to answer these questions they were excluded. After exclusion 17 698 participants remained (81% of 21 838). The study was approved by the University Medical Centre Utrecht medical ethical commission and all participants gave online informed consent. Of these 17698 participants, 1259 provided genetic information, via two waves.

**Quality control:**

*IMAGEN*

SNPs with a minor allele frequency (MAF) of less than 2%, a genotyping rate of 2% or SNPs that did not respect Hardy Weinberg Equilibrium (HWE) (<1x10^-6^^-6^) were removed from analysis. Individuals with disproportionate levels of individual missingness (<2%), ambiguous sex, evidence of cryptic relatedness (>0.125), excessive heterozygosity were removed. After the first quality control a total of 488426 SNPs remained. The SNP coordinates were updated from hg18 to hg19 using Illumina information and the liftover tool from the genome browser. (<http://genome.ucsc.edu/cgi-bin/hgLiftOver)>. After the first steps of quality control, 1950 individuals remained. The data of the IMAGEN cohort were then combined with data from HapMap III, and principal component analysis was performed in PLINK1.9[3] to determine ancestry information. We removed individuals who did not fall within 3SD of the mean of the first 2 principal components of the CEU + TSI populations from HapMapIII[4]. In all, 1740 individuals remained for polygenic risk score and regression analysis.

*CannabisQuest*

DNA was extracted from whole blood of two 10 ml EDTA tubes obtained using venipuncture. For logistic reasons, genotype data for individuals of Dutch ancestry were generated on two different array platforms; 576 individuals on Illumina® HumanOmniExpress (733,202 SNPs) and 768 individuals on the Illumina® Human610-Quad Beadchip (620,901 SNPs). In this cohort, participants were excluded based on errors in sex and on > 5% missing genotypes. SNPs were filtered on missingness, MAF, and HWE, using the same criteria as the IMAGEN dataset. The CannabisQuest cohort genetic dataset was also imputed, as described in Boks et al. 202 [2], using the Hapmap III release 24 using Beagle [5].

**Base Data Set**

*CLOSUK + PGS (Pardiñas et al. 2018)*  [6]

To build polygenic risk score for schizophrenia (PRS-Sz) we use a most recent and largest schizophrenia GWAS to date [6] as a training set. The summary statistics used for PRS construction came from a meta-analysis of the CLOZUK sample (treatment resistant schizophrenia) and independent psychiatric genomics consortium (PGC) datasets (schizophrenia) (total 40,675 cases and 64,643 control). The CLOZUK sample was demonstrably similar to previous PGC schizophrenia sample[6]. The entirety of the sample (meta-analysis results) was included for construction of PRS, as it was determined that the CLOZUK + PGC sample was independent and non-overlapping to the IMAGEN and CannabisQuest samples.

*Cannabis Use (Pasman et al., 2018) [7]*

We use the publicly available GWAS meta-analysis results of from the International Cannabis Consortium (ICC) for the creation of the cannabis use polygenic risk score. The ICC GWAS separated cases and controls as a binary lifetime measure of cannabis use, “yes” or “no”. The Utrecht cohort data contributed to this GWAS, and as such, a cannabis use PRS was not constructed for the Utrecht cohort: a leave-one out dataset was not available. Although data from a 23andMe cohort is available online for use with the ICC cannabis cohort (PGC), we did not have data use agreements in place, and thus the did not include this data into our PGC calculations. Overall, data for PRS construction was from 162 082 individuals.

***Polygenic Risk Score***

Polygenic Risk Scores for schizophrenia (PRS-Sz) were constructed for each IMAGEN and Utrecht individuals based on data from the most recent schizophrenia GWAS based on 40 675 cases and 64 643 controls [6] as a training set. Prior to PRS building, SNPs from the base set were removed if they had an MAF <0.01. Overlapping SNPs between the GWAS summary statistics (base dataset), 1000 reference Genome (reference dataset), and our dataset (target dataset; IMAGEN or Utrecht) were selected. Then 1) insertion or deletion, ambiguous SNPs, 2) SNPs with minor allele frequency (MAF)<0.01 and SNP with imputation quality (R2) < 0.8 in both training dataset and target dataset 3) SNPs located in complex-LD regions (supplementary table 1) were excluded[8]. PRScs is then used to infer posterior SNP effect sizes, by placing a continuous shrinkage prior on SNP effect sizes reported in the most recent schizophrenia GWAS (Ge et al. 2019), as well as an external LD reference panel. As described in their article, PRScs utilizes a Bayesian regression framework, which allows for “multivariate modeling of local LD patterns” (Ge et al. 2019). PRS calculations using PRScs posterior effect sizes have been shown to outperform many existing methods (Ge et al. 2019; Pain et al. 2021). In our work, we use the 1000 Genomes Project European LD reference panel provided here (<https://github.com/getian107/PRScs>). We use the default settings available in the PRScs script as described on <https://github.com/getian107/PRScs/>

The PRS for cannabis use (PRS-CanUse) was constructed similarly to previously published methods [9]. While the Johnson group used PLINK1.9[3] *clump* and *score* procedures, we created PRS using comparable methods in PRSice2[10]. Clumping was however with respect to 1000 Genome Phase 3 european samples, as it was done in the Johnson work. Moreover a 500kb physicial distance and LD threshold of r^2^>=0.25 were used. We calculated PRS for 12 GWAS p-value thresholds: 5 x 10-8, 5 x 10-7, 5 x 10-6, 5 x 10-5, 5 x 10-4, 5 x 10-3, 0.05, 0.1, 0.2, 0.3, 0.4 and 0.5. Considering that PRS-Can use was not predictive of cannabis use at 16 years of age, we calculated PRS for cannabis users at 18 years of age in the IMAGEN cohort. The PRS most predictive of case/control (>10 lifetime cannabis uses), was Pt = 0.05, and this PRS was therefore used as a covariable in sensitivity analyses.

**Bibliography**

1. Schumann G, Loth E, Banaschewski T, Barbot A, Barker G, Büchel C, et al. The IMAGEN study: reinforcement-related behaviour in normal brain function and psychopathology. Mol Psychiatry. 2010;15:1128–1139.

2. Boks MP, He Y, Schubart CD, Gastel W van, Elkrief L, Huguet G, et al. Cannabinoids and psychotic symptoms: A potential role for a genetic variant in the P2X purinoceptor 7 (P2RX7) gene. Brain, Behavior, and Immunity. 2020;88:573–581.

3. Chang CC, Chow CC, Tellier LC, Vattikuti S, Purcell SM, Lee JJ. Second-generation PLINK: rising to the challenge of larger and richer datasets. Gigascience. 2015;4.

4. Altshuler DM, Gibbs RA, Peltonen L, Altshuler DM, Gibbs RA, Peltonen L, et al. Integrating common and rare genetic variation in diverse human populations. Nature. 2010;467:52–58.

5. Browning BL, Browning SR. A Unified Approach to Genotype Imputation and Haplotype-Phase Inference for Large Data Sets of Trios and Unrelated Individuals. Am J Hum Genet. 2009;84:210–223.

6. Pardiñas AF, Holmans P, Pocklington AJ, Escott-Price V, Ripke S, Carrera N, et al. Common schizophrenia alleles are enriched in mutation-intolerant genes and in regions under strong background selection. Nat Genet. 2018;50:381–389.

7. Pasman JA, Verweij KJH, Gerring Z, Stringer S, Sanchez-Roige S, Treur JL, et al. GWAS of lifetime cannabis use reveals new risk loci, genetic overlap with psychiatric traits, and a causal influence of schizophrenia. Nat Neurosci. 2018;21:1161–1170.

8. Price AL, Weale ME, Patterson N, Myers SR, Need AC, Shianna KV, et al. Long-Range LD Can Confound Genome Scans in Admixed Populations. Am J Hum Genet. 2008;83:132–135.

9. Johnson EC, Tillman R, Aliev F, Meyers JL, Salvatore JE, Anokhin AP, et al. Exploring the relationship between polygenic risk for cannabis use, peer cannabis use and the longitudinal course of cannabis involvement. Addiction. 2019;114:687–697.

10. Choi SW, O’Reilly PF. PRSice-2: Polygenic Risk Score software for biobank-scale data. Gigascience. 2019;8.

**Supplementary Tables**

**Supplementary Table 1. The complex-LD regions (build GRCh37) were removed before PRS calculation.**

| Chromosome | Base pair start | Base pair end |
| --- | --- | --- |
| 6 | 25392021 | 33392022 |
| 8 | 111930824 | 114930824 |
| 11 | 46043424 | 57243424 |
| 1 | 48287980 | 52287979 |
| 2 | 86088342 | 101041482 |
| 2 | 134666268 | 138166268 |
| 2 | 183174494 | 190174494 |
| 3 | 47524996 | 50024996 |
| 3 | 83417310 | 86917310 |
| 3 | 88917310 | 96017310 |
| 5 | 44464243 | 50464243 |
| 5 | 97972100 | 100472101 |
| 5 | 128972101 | 131972101 |
| 5 | 135472101 | 138472101 |
| 6 | 56892041 | 63942041 |
| 6 | 139958307 | 142458307 |
| 7 | 55225791 | 66555850 |
| 8 | 7962590 | 11962591 |
| 8 | 42880843 | 49837447 |
| 10 | 36959994 | 43679994 |
| 11 | 87860352 | 90860352 |
| 12 | 33108733 | 41713733 |
| 12 | 111037280 | 113537280 |
| 20 | 32536339 | 35066586 |

**Supplementary Table S2. Sample Characteristics**

|  |  | | Female (N=880) | Male (N=860) | Total (N=1740) | p value |
| --- | --- | --- | --- | --- | --- | --- |
| IMAGEN |  |  | | |  |  |
| Cannabis Use |  |  |  |  |  | < 0.001 |
|  | Mean (SD) |  | 0.636 (1.430) | 1.034 (1.878) | 0.829 (1.675) |  |
|  | Min - Max |  | 0 - 6 | 0 - 6 | 0- 6 |  |
|  | Missing |  | 158 | 175 | 333 |  |
| Positive Dimension | |  |  |  |  |  |
|  | Mean (SD) |  | 24.79 (4.22) | 24.82 (4.52) | 24.8 (4.36) | 0.8 |
|  | Min - Max |  | 19 - 54 | 19 - 55 | 19 - 55 |  |
|  | Missing |  | 269 | 315 | 584 |  |
| Depressive Dimension | |  |  |  |  |  |
|  | Mean (SD) |  | 14.3 (3.81) | 12.77 (3.1) | 13.58 (3.57) | < 0.001 |
|  | Min - Max |  | 8 – 30 | 8 – 28 | 8 – 30 |  |
|  | Missing |  | 269 | 315 | 584 |  |
| Negative Dimension | |  |  |  |  |  |
|  | Mean (SD) |  | 22.46 (5.61) | 22.05 (5.61) | 22.265 (5.61) | 0.2 |
|  | Min - Max |  | 14 - 45 | 14 - 44 | 14 – 45 |  |
|  | Missing |  | 269 | 315 | 584 |  |
| Total CAPE-42 |  |  |  |  |  |  |
|  | Mean (SD) |  | 61.54 (11.65) | 59.64 (11.32) | 60.65 (11.53) | 0.005 |
|  | Min - Max |  | 41 - 115 | 41 - 121 | 41 - 121 |  |
|  | Missing |  | 269 | 315 | 584 |  |
|  |  |  |  |  |  |  |
| Utrecht cannabis | |  | **Female (N=658)** | **Male(N=565)** | **Total(N=1223)** | p value |
| Age |  |  |  |  |  |  |
|  | Mean (SD) |  | 20.34 (2.39) | 20.58 (2.46) | 20.45 (2.42) | 0.08 |
|  | Min - Max |  | 16 - 40 | 16 - 39 | 16 – 40 |  |
|  | Missing |  | 0 | 0 | 0 |  |
| Cannabis Use |  |  |  |  |  | < 0.001 |
|  | Mean (SD) |  | 1.117 (1.653) | 2.558 (1.842) | 1.783 (1.885) |  |
|  | Min - Max |  | 0.000 - 4.000 | 0.000 - 4.000 | 0.000 - 4.000 |  |
|  | Missing |  | 0 | 0 | 0 |  |
| CAPE42 |  |  |  |  |  |  |
| Positive Dimension | |  |  |  |  |  |
|  | Mean (SD) |  | 28.24 (8.32) | 28.01 (5.41) | 28.13 (7.12) | 0.6 |
|  | Min - Max |  | 20 – 175 | 20 – 49 | 20 - 175 |  |
|  | Missing |  | 0 | 0 | 0 |  |
| Depressive Dimension | |  |  |  |  |  |
|  | Mean (SD) |  | 14.45 (3.67) | 13.09 (3.32) | 13.82 (3.58) | < 0.001 |
|  | Min - Max |  | 8 - 40 | 8 – 28 | 8 - 40 |  |
|  | Missing |  | 0 | 0 | 0 |  |
| Negative Dimension | |  |  |  |  |  |
|  | Mean (SD) |  | 25.42 (6.61) | 25.93 (6.93) | 25.65 (6.76) | 0.2 |
|  | Min - Max |  | 15 - 58 | 15 - 47 | 15 - 58 |  |
|  | Missing |  | 0 | 0 | 0 |  |
| Total CAPE-42 |  |  |  |  |  |  |
|  | Mean (SD) |  | 67.77 (14.31) | 67.03 (13.59) | 67.43 (13.98) | 0.4 |
|  | Min - Max |  | 23 - 146 | 43 - 110 | 23 – 146 |  |
|  | Missing |  | 0 | 0 | 0 |  |

Supplementary Table S2. Summary statistics. T-test are performed to determine difference of means between male and female subjects. Missing = number of individuals with missing phenotype data.

**Supplementary Table S3. Predictive value of PRS-Sz on PLE (Model 1)**

| IMAGEN | β | std.error | statistic | p |
| --- | --- | --- | --- | --- |
| PRS-Sz | 0.015 | 0.005 | 2.85 | ***4.43 × 10^−3^*** |
| SEX | 0.030 | 0.010 | 2.85 | ***4.49 × 10^−3^*** |
| PC1 | 0.006 | 0.007 | 0.77 | 4.41 × 10^−1^ |
| PC2 | −0.0038 | 0.005 | −0.71 | 4.76 × 10^−1^ |
| PC3 | −0.011 | 0.007 | −1.49 | 1.37 × 10^−1^ |
| PC4 | −0.0066 | 0.007 | −0.94 | 3.48 × 10^−1^ |
| PC5 | −0.0024 | 0.005 | −0.45 | 6.54 × 10^−1^ |
| PC6 | 0.005 | 0.010 | 0.49 | 6.27 × 10^−1^ |
|  |  |  |  |  |
| Utrecht cannabis |  |  |  |  |
| PRS-Sz | 0.021 | 0.006 | 3.62 | ***3.11 × 10^−4^*** |
| SEX | 0.010 | 0.011 | 0.87 | 3.82 × 10^−1^ |
| AGE | −0.0012 | 0.002 | −0.50 | 6.20 × 10^−1^ |
| PC1 | 0.007 | 0.006 | 1.29 | 1.98 × 10^−1^ |
| PC2 | 0.011 | 0.006 | 1.86 | 6.29 × 10^−2^ |
| PC3 | 0.000 | 0.006 | 0.02 | 9.81 × 10^−1^ |
| PC4 | −0.0053 | 0.006 | −0.93 | 3.53 × 10^−1^ |
| PC5 | −0.0043 | 0.006 | −0.75 | 4.54 × 10^−1^ |
| PC6 | −0.0019 | 0.006 | −0.36 | 7.37 × 10^−1^ |

**Supplementary Table S3.** Predictive value of PRS-Sz on PLE. We include the first 6 PC and Sex as covariates for all analyses and age is included for all analyses of the Utrecht cohort. β= Beta, std.error = standard error, p = p value. PRS-Sz = polygenic risk score for schizophrenia. pt=p-value threshold.

**Supplementary Table S4. Predictive value of schizophrenia polygenic risk score on cannabis use (Model 2)**

| IMAGEN | β | std.error | statistic | p |
| --- | --- | --- | --- | --- |
| PRS-Sz | 0.097 | 0.044 | 2.21 | ***2.72 × 10^−2^*** |
| SEX | −0.41 | 0.088 | −4.65 | ***3.66 × 10^−6^*** |
| PC1 | 0.037 | 0.063 | 0.60 | 5.51 × 10^−1^ |
| PC2 | −0.021 | 0.045 | −0.46 | 6.45 × 10^−1^ |
| PC3 | 0.014 | 0.060 | 0.23 | 8.15 × 10^−1^ |
| PC4 | 0.117 | 0.059 | 1.98 | ***4.80 × 10^−2^*** |
| PC5 | −0.022 | 0.045 | −0.49 | 6.24 × 10^−1^ |
| PC6 | −0.20 | 0.083 | −2.38 | ***1.76 × 10^−2^*** |
|  |  |  |  |  |
| Utrecht cannabis |  |  |  |  |
| PRS-Sz | 0.24 | 0.049 | 4.76 | ***2.21 × 10^−6^*** |
| SEX | −1.42 | 0.099 | −14.39 | ***1.83 × 10^−43^*** |
| AGE | 0.048 | 0.020 | 2.37 | ***1.80 × 10^−2^*** |
| PC1 | −0.10 | 0.049 | −2.09 | ***3.66 × 10^−2^*** |
| PC2 | 0.009 | 0.049 | 0.18 | 8.58 × 10^−1^ |
| PC3 | −0.014 | 0.049 | −0.29 | 7.76 × 10^−1^ |
| PC4 | −0.011 | 0.049 | −0.23 | 8.22 × 10^−1^ |
| PC5 | −0.094 | 0.049 | −1.91 | 5.60 × 10^−2^ |
| PC6 | −0.042 | 0.049 | −0.85 | 3.97 × 10^−1^ |

**Supplementary Table S4.** Predictive value of PRS-Sz on cannabis use. Above are results from the linear regression measuring predictive value of PRS-Sz (independent variable) and cannabis use (dependent variable). We include the first 6 PC and Sex as covariates for all analyses and age is included for all analyses of the CannabisQuest cohort. . β= Beta, std.error = standard error, p = p value. PRS-Sz = polygenic risk score for schizophrenia. pt=p-value threshold.

**Supplementary Table S5. Predictive value of PRS-Sz and cannabis use on psychotic-like experiences (Model 3)**

| IMAGEN | β | std.error | statistic | p |
| --- | --- | --- | --- | --- |
| PRS-Sz | 0.014 | 0.0055 | 2.57 | ***1.03 × 10^−2^*** |
| Cannabis use | 0.009 | 0.0034 | 2.71 | ***6.91 × 10^−3^*** |
| SEX | 0.036 | 0.0110 | 3.25 | ***1.18 × 10^−3^*** |
| PC1 | 0.006 | 0.0077 | 0.78 | 4.37 × 10^−1^ |
| PC2 | −0.0031 | 0.0056 | −0.55 | 5.81 × 10^−1^ |
| PC3 | −0.0097 | 0.0074 | −1.31 | 1.90 × 10^−1^ |
| PC4 | −0.0066 | 0.0072 | −0.91 | 3.62 × 10^−1^ |
| PC5 | −0.0018 | 0.0056 | −0.36 | 7.45 × 10^−1^ |
| PC6 | 0.009 | 0.0100 | 0.94 | 3.47 × 10^−1^ |
| Utrecht cannabis |  |  |  |  |
| PRS-Sz | 0.017 | 0.0057 | 2.93 | ***3.48 × 10^−3^*** |
| Cannabis use | 0.017 | 0.0033 | 5.12 | ***3.61 × 10^−7^*** |
| SEX | 0.034 | 0.0122 | 2.77 | ***5.69 × 10^−3^*** |
| AGE | −0.0020 | 0.0023 | −0.85 | 3.97 × 10^−1^ |
| PC1 | 0.009 | 0.0057 | 1.61 | 1.09 × 10^−1^ |
| PC2 | 0.010 | 0.0056 | 1.85 | 6.39 × 10^−2^ |
| PC3 | 0.000 | 0.0057 | 0.07 | 9.48 × 10^−1^ |
| PC4 | −0.0051 | 0.0056 | −0.91 | 3.66 × 10^−1^ |
| PC5 | −0.0027 | 0.0056 | −0.48 | 6.35 × 10^−1^ |
| PC6 | −0.0012 | 0.0056 | −0.22 | 8.30 × 10^−1^ |

**Supplementary Table S5.** Predictive value of PRS-Sz and cannabis use on psychotic-like experiences. Above are results from the linear regression measuring predictive value of PRS-Sz (independent variable), cannabis use (independent variable) and cannabis use (dependent variable). We include the first 6 PC and sex as covariates for all analyses and age is included for all analyses of the Utrecht cannabis cohort. β= Beta, std.error = standard error, p = p value. PRS-Sz = polygenic risk score for schizophrenia. pt=p-value threshold.

**Supplementary Table S6 Full moderation analysis**

| IMAGEN | β | std.error | statistic | p |
| --- | --- | --- | --- | --- |
| PRS-Sz | 0.016 | 0.0061 | 2.55 | ***1.09 × 10^−2^*** |
| Cannabis use | 0.009 | 0.0034 | 2.74 | ***6.29 × 10^−3^*** |
| SEX | 0.036 | 0.0110 | 3.27 | ***1.09 × 10^−3^*** |
| PC1 | 0.006 | 0.0077 | 0.77 | 4.43 × 10^−1^ |
| PC2 | −0.0030 | 0.0056 | −0.53 | 5.94 × 10^−1^ |
| PC3 | −0.0098 | 0.0074 | −1.32 | 1.87 × 10^−1^ |
| PC4 | −0.0063 | 0.0073 | −0.87 | 3.86 × 10^−1^ |
| PC5 | −0.0018 | 0.0056 | −0.32 | 7.52 × 10^−1^ |
| PC6 | 0.010 | 0.0101 | 0.98 | 3.29 × 10^−1^ |
| PRS:Cannabis | −0.0020 | 0.0036 | −0.56 | 5.78 × 10^−1^ |
| Utrecht cannabis | |  |  |  |
| PRS-Sz | 0.017 | 0.0082 | 2.04 | ***4.13 × 10^−2^*** |
| Cannabis use | 0.017 | 0.0033 | 5.11 | ***3.66 × 10^−7^*** |
| SEX | 0.034 | 0.012 | 2.77 | ***5.74 × 10^−3^*** |
| AGE | −0.0020 | 0.0023 | −0.85 | 3.97 × 10^−1^ |
| PC1 | 0.009 | 0.0057 | 1.61 | 1.09 × 10^−1^ |
| PC2 | 0.010 | 0.0056 | 1.85 | 6.43 × 10^−2^ |
| PC3 | 0.000 | 0.0057 | 0.07 | 9.48 × 10^−1^ |
| PC4 | −0.0051 | 0.0056 | −0.91 | 3.66 × 10^−1^ |
| PC5 | −0.0027 | 0.0056 | −0.48 | 6.35 × 10^−1^ |
| PC6 | −0.0012 | 0.0056 | −0.22 | 8.29 × 10^−1^ |
| PRS:Cannabis | −0.000044 | 0.0030 | −0.015 | 9.88 × 10^−1^ |

**Supplementary Table S6.** Full Moderation Analysis. We include the first 6 PC and Sex as covariates for all analyses and age is included for all analyses of the Utrecht cohort.

β= Beta, std.error = standard error, p = p value. PRS-Sz = polygenic risk score for schizophrenia. pt=p-value threshold.

**Supplementary Table S7.** Full mediation analysis.

| **Dependent Variable** |  | **Independent Variable** | **label** | β | **se** | **z** | **pvalue** | **ci.lower** | **ci.upper** |
| --- | --- | --- | --- | --- | --- | --- | --- | --- | --- |
| CAPE | ~ | PRS | c | 0.0141 | 0.0052 | 2.6981 | 0.0070 | 0.0038 | 0.0243 |
| CAPE | ~ | Cannabis | b | 0.0091 | 0.0033 | 2.7207 | 0.0065 | 0.0025 | 0.0156 |
| CAPE | ~ | Sex |  | 0.0333 | 0.0105 | 3.1850 | 0.0014 | 0.0128 | 0.0538 |
| CAPE | ~ | PC1 |  | 0.0055 | 0.0074 | 0.7437 | 0.4570 | -0.0090 | 0.0200 |
| CAPE | ~ | PC2 |  | -0.0034 | 0.0053 | -0.6314 | 0.5278 | -0.0139 | 0.0071 |
| CAPE | ~ | PC3 |  | -0.0101 | 0.0070 | -1.4398 | 0.1499 | -0.0239 | 0.0037 |
| CAPE | ~ | PC4 |  | -0.0072 | 0.0069 | -1.0421 | 0.2974 | -0.0208 | 0.0064 |
| CAPE | ~ | PC5 |  | -0.0016 | 0.0054 | -0.3074 | 0.7585 | -0.0122 | 0.0089 |
| CAPE | ~ | PC6 |  | 0.0063 | 0.0095 | 0.6616 | 0.5082 | -0.0123 | 0.0249 |
| Cannabis | ~ | PRS | a | 0.0968 | 0.0437 | 2.2168 | 0.0266 | 0.0112 | 0.1824 |
| Cannabis | ~ | Sex |  | -0.4098 | 0.0876 | -4.6787 | 0.0000 | -0.5814 | -0.2381 |
| Cannabis | ~ | PC1 |  | 0.0370 | 0.0624 | 0.5930 | 0.5532 | -0.0853 | 0.1594 |
| Cannabis | ~ | PC2 |  | -0.0211 | 0.0451 | -0.4670 | 0.6405 | -0.1095 | 0.0673 |
| Cannabis | ~ | PC3 |  | 0.0138 | 0.0600 | 0.2308 | 0.8175 | -0.1038 | 0.1315 |
| Cannabis | ~ | PC4 |  | 0.1164 | 0.0588 | 1.9795 | 0.0478 | 0.0011 | 0.2317 |
| Cannabis | ~ | PC5 |  | -0.0220 | 0.0448 | -0.4904 | 0.6239 | -0.1097 | 0.0658 |
| Cannabis | ~ | PC6 |  | -0.1986 | 0.0823 | -2.4119 | 0.0159 | -0.3599 | -0.0372 |
| CAPE | ~~ | CAPE |  | 0.0309 | 0.0013 | 24.0261 | 0.0000 | 0.0283 | 0.0334 |
| Cannabis | ~~ | Cannabis |  | 2.6862 | 0.1013 | 26.5234 | 0.0000 | 2.4877 | 2.8847 |
| PRS | ~~ | PRS |  | 0.9994 | 0.0000 | NA | NA | 0.9994 | 0.9994 |
| PRS | ~~ | Sex |  | 0.0107 | 0.0000 | NA | NA | 0.0107 | 0.0107 |
| PRS | ~~ | PC1 |  | -0.0523 | 0.0000 | NA | NA | -0.0523 | -0.0523 |
| PRS | ~~ | PC2 |  | 0.0346 | 0.0000 | NA | NA | 0.0346 | 0.0346 |
| PRS | ~~ | PC3 |  | -0.0319 | 0.0000 | NA | NA | -0.0319 | -0.0319 |
| PRS | ~~ | PC4 |  | -0.0066 | 0.0000 | NA | NA | -0.0066 | -0.0066 |
| PRS | ~~ | PC5 |  | -0.0044 | 0.0000 | NA | NA | -0.0044 | -0.0044 |
| PRS | ~~ | PC6 |  | -0.0023 | 0.0000 | NA | NA | -0.0023 | -0.0023 |
| Sex | ~~ | Sex |  | 0.2500 | 0.0000 | NA | NA | 0.2500 | 0.2500 |
| Sex | ~~ | PC1 |  | -0.0132 | 0.0000 | NA | NA | -0.0132 | -0.0132 |
| Sex | ~~ | PC2 |  | -0.0066 | 0.0000 | NA | NA | -0.0066 | -0.0066 |
| Sex | ~~ | PC3 |  | -0.0123 | 0.0000 | NA | NA | -0.0123 | -0.0123 |
| Sex | ~~ | PC4 |  | 0.0039 | 0.0000 | NA | NA | 0.0039 | 0.0039 |
| Sex | ~~ | PC5 |  | 0.0133 | 0.0000 | NA | NA | 0.0133 | 0.0133 |
| Sex | ~~ | PC6 |  | -0.0035 | 0.0000 | NA | NA | -0.0035 | -0.0035 |
| PC1 | ~~ | PC1 |  | 0.9994 | 0.0000 | NA | NA | 0.9994 | 0.9994 |
| PC1 | ~~ | PC2 |  | -0.0825 | 0.0000 | NA | NA | -0.0825 | -0.0825 |
| PC1 | ~~ | PC3 |  | 0.5587 | 0.0000 | NA | NA | 0.5587 | 0.5587 |
| PC1 | ~~ | PC4 |  | -0.4443 | 0.0000 | NA | NA | -0.4443 | -0.4443 |
| PC1 | ~~ | PC5 |  | 0.1180 | 0.0000 | NA | NA | 0.1180 | 0.1180 |
| PC1 | ~~ | PC6 |  | 0.6706 | 0.0000 | NA | NA | 0.6706 | 0.6706 |
| PC2 | ~~ | PC2 |  | 0.9994 | 0.0000 | NA | NA | 0.9994 | 0.9994 |
| PC2 | ~~ | PC3 |  | -0.0920 | 0.0000 | NA | NA | -0.0920 | -0.0920 |
| PC2 | ~~ | PC4 |  | -0.1418 | 0.0000 | NA | NA | -0.1418 | -0.1418 |
| PC2 | ~~ | PC5 |  | 0.0366 | 0.0000 | NA | NA | 0.0366 | 0.0366 |
| PC2 | ~~ | PC6 |  | 0.0813 | 0.0000 | NA | NA | 0.0813 | 0.0813 |
| PC3 | ~~ | PC3 |  | 0.9994 | 0.0000 | NA | NA | 0.9994 | 0.9994 |
| PC3 | ~~ | PC4 |  | -0.3726 | 0.0000 | NA | NA | -0.3726 | -0.3726 |
| PC3 | ~~ | PC5 |  | 0.0475 | 0.0000 | NA | NA | 0.0475 | 0.0475 |
| PC3 | ~~ | PC6 |  | 0.6386 | 0.0000 | NA | NA | 0.6386 | 0.6386 |
| PC4 | ~~ | PC4 |  | 0.9994 | 0.0000 | NA | NA | 0.9994 | 0.9994 |
| PC4 | ~~ | PC5 |  | -0.1095 | 0.0000 | NA | NA | -0.1095 | -0.1095 |
| PC4 | ~~ | PC6 |  | -0.6702 | 0.0000 | NA | NA | -0.6702 | -0.6702 |
| PC5 | ~~ | PC5 |  | 0.9994 | 0.0000 | NA | NA | 0.9994 | 0.9994 |
| PC5 | ~~ | PC6 |  | 0.2059 | 0.0000 | NA | NA | 0.2059 | 0.2059 |
| PC6 | ~~ | PC6 |  | 0.9994 | 0.0000 | NA | NA | 0.9994 | 0.9994 |
| CAPE | ~1 |  |  | 4.0312 | 0.0174 | 232.2638 | 0.0000 | 3.9972 | 4.0652 |
| Cannabis | ~1 |  |  | 1.4578 | 0.1396 | 10.4445 | 0.0000 | 1.1842 | 1.7314 |
| PRS | ~1 |  |  | 0.0000 | 0.0000 | NA | NA | 0.0000 | 0.0000 |
| Sex | ~1 |  |  | 1.5057 | 0.0000 | NA | NA | 1.5057 | 1.5057 |
| PC1 | ~1 |  |  | 0.0000 | 0.0000 | NA | NA | 0.0000 | 0.0000 |
| PC2 | ~1 |  |  | 0.0000 | 0.0000 | NA | NA | 0.0000 | 0.0000 |
| PC3 | ~1 |  |  | 0.0000 | 0.0000 | NA | NA | 0.0000 | 0.0000 |
| PC4 | ~1 |  |  | 0.0000 | 0.0000 | NA | NA | 0.0000 | 0.0000 |
| PC5 | ~1 |  |  | 0.0000 | 0.0000 | NA | NA | 0.0000 | 0.0000 |
| PC6 | ~1 |  |  | 0.0000 | 0.0000 | NA | NA | 0.0000 | 0.0000 |
| Cannabis_IDE | := | a*b | Cannabis_IDE | 0.0009 | 0.0005 | 1.7190 | 0.0856 | -0.0001 | 0.0019 |
| total | := | c+(a*b) | total | 0.0149 | 0.0052 | 2.8652 | 0.0042 | 0.0047 | 0.0252 |

**Supplementary Table S7.** Full results of mediation analysis. We include the first 6 PC and sex as covariates for all analyses and age is included for all analyses of the Utrecht cannabis cohort. β= Beta, std.error = standard error, p = p value. PRS-Sz = polygenic risk score for schizophrenia. pt=p-value threshold.

**Supplementary Table S8.** Predictive value of SCZ polygenic risk scores (PRS) on CAPE-42 subscales in IMAGEN cohort.

|  | Independent Variable | β | std.error | statistic | p |
| --- | --- | --- | --- | --- | --- |
| Depressive sub-scale |  |  |  |  |  |
|  | PRS-Sz | 0.0184 | 0.0072 | 2.5420 | ***1.12 × 10^−2^*** |
|  | Cannabis Use | 0.0100 | 0.0044 | 2.2491 | ***2.47 × 10^−2^*** |
|  | SEX | 0.1121 | 0.0145 | 7.7187 | ***2.72 × 10^−14^*** |
|  | PC1 | 0.0062 | 0.0103 | 0.6084 | 5.43 × 10^−1^ |
|  | PC2 | −0.0033 | 0.0074 | −0.45 | 6.53 × 10^−1^ |
|  | PC3 | −0.0059 | 0.0098 | −0.60 | 5.47 × 10^−1^ |
|  | PC4 | −0.0076 | 0.0096 | −0.79 | 4.27 × 10^−1^ |
|  | PC5 | −0.0065 | 0.0074 | −0.87 | 3.83 × 10^−1^ |
|  | PC6 | −0.0033 | 0.0133 | −0.25 | 8.05 × 10^−1^ |
|  |  |  |  |  |  |
| Negative sub-scale |  |  |  |  |  |
|  | PRS-Sz | 0.0144 | 0.0074 | 1.9385 | 5.28 × 10^−2^ |
|  | Cannabis Use | 0.0132 | 0.0046 | 2.8977 | ***3.84 × 10^−3^*** |
|  | SEX | 0.0256 | 0.0149 | 1.7203 | 8.57 × 10^−2^ |
|  | PC1 | 0.0111 | 0.0105 | 1.0586 | 2.90 × 10^−1^ |
|  | PC2 | −0.0036 | 0.0075 | −0.48 | 6.35 × 10^−1^ |
|  | PC3 | −0.0045 | 0.0100 | −0.44 | 6.57 × 10^−1^ |
|  | PC4 | −0.017 | 0.0098 | −1.69 | 9.05 × 10^−2^ |
|  | PC5 | −0.0058 | 0.0076 | −0.77 | 4.40 × 10^−1^ |
|  | PC6 | −0.00098 | 0.0136 | −0.072 | 9.43 × 10^−1^ |
|  |  |  |  |  |  |
| Positive sub-scale |  |  |  |  |  |
|  | PRS-Sz | 0.0107 | 0.0049 | 2.2059 | ***2.76 × 10^−2^*** |
|  | Cannabis Use | 0.0041 | 0.0030 | 1.3793 | 1.68 × 10^−1^ |
|  | SEX | 0.0034 | 0.0098 | 0.3495 | 7.27 × 10^−1^ |
|  | PC1 | 0.0022 | 0.0069 | 0.3143 | 7.53 × 10^−1^ |
|  | PC2 | −0.0012 | 0.0049 | −0.23 | 8.15 × 10^−1^ |
|  | PC3 | −0.016 | 0.0066 | −2.39 | ***1.69 × 10^−2^*** |
|  | PC4 | 0.0032 | 0.0065 | 0.4931 | 6.22 × 10^−1^ |
|  | PC5 | 0.0035 | 0.0050 | 0.7071 | 4.80 × 10^−1^ |
|  | PC6 | 0.0254 | 0.0089 | 2.8380 | ***4.63 × 10^−3^*** |

**Supplementary Table S8.** Predictive value of PRS-Sz and Cannabis on CAPE-42 subscales, in IMAGEN cohort. This table shows results for the linear regressions of various schizophrenia polygenic risk score on CAPE-42 subscales. We include the first 6 PC and sex as covariates for all analyses and age is included for all analyses of the CannabisQuest cohort. β= Beta, std.error = standard error, p = p value. PRS-Sz = polygenic risk score for schizophrenia. pt=p-value threshold.

Supplementary Table S9. Predictive value of PRS-Sz and Cannabis on CAPE-42 subscales, in Utrecht cohort.

|  | Independent Variable | β | std.error | statistic | p |
| --- | --- | --- | --- | --- | --- |
| Depressive sub-scale | |  |  |  |  |
|  | PRS-Sz | 0.022 | 0.007 | 3.272 | ***1.10 × 10^−3^*** |
|  | Cannabis Use | 0.010 | 0.004 | 2.693 | ***7.17 × 10^−3^*** |
|  | SEX | 0.116 | 0.014 | 8.001 | ***2.86 × 10^−15^*** |
|  | AGE | 0.004 | 0.003 | 1.389 | 1.65 × 10^−1^ |
|  | PC1 | 0.001 | 0.007 | 0.189 | 8.50 × 10^−1^ |
|  | PC2 | 0.012 | 0.007 | 1.854 | 6.40 × 10^−2^ |
|  | PC3 | −0.0059 | 0.007 | −0.89 | 3.74 × 10^−1^ |
|  | PC4 | −0.0081 | 0.007 | −1.23 | 2.22 × 10^−1^ |
|  | PC5 | −0.0067 | 0.007 | −1.01 | 3.13 × 10^−1^ |
|  | PC6 | −0.000001 | 0.007 | −0.00012 | 1.00 × 10^0^ |
| Negative sub-scale | |  |  |  |  |
|  | PRS-Sz | 0.023 | 0.007 | 3.121 | ***1.85 × 10^−3^*** |
|  | Cannabis Use | 0.019 | 0.004 | 4.508 | ***7.17 × 10^−6^*** |
|  | SEX | 0.011 | 0.016 | 0.728 | 4.67 × 10^−1^ |
|  | AGE | 0.003 | 0.003 | 0.986 | 3.24 × 10^−1^ |
|  | PC1 | 0.013 | 0.007 | 1.802 | 7.18 × 10^−2^ |
|  | PC2 | 0.009 | 0.007 | 1.316 | 1.88 × 10^−1^ |
|  | PC3 | 0.002 | 0.007 | 0.301 | 7.63 × 10^−1^ |
|  | PC4 | −0.0045 | 0.007 | −0.62 | 5.36 × 10^−1^ |
|  | PC5 | −0.0037 | 0.007 | −0.52 | 6.05 × 10^−1^ |
|  | PC6 | 0.001 | 0.007 | 0.185 | 8.53 × 10^−1^ |
| Positive sub-scale | |  |  |  |  |
|  | PRS-Sz | 0.011 | 0.006 | 1.891 | 5.88 × 10^−2^ |
|  | Cannabis Use | 0.017 | 0.003 | 5.374 | ***9.24 × 10^−8^*** |
|  | SEX | 0.024 | 0.012 | 1.970 | ***4.91 × 10^−2^*** |
|  | AGE | −0.0079 | 0.002 | −3.44 | ***6.02 × 10^−4^*** |
|  | PC1 | 0.005 | 0.006 | 0.938 | 3.49 × 10^−1^ |
|  | PC2 | 0.008 | 0.006 | 1.376 | 1.69 × 10^−1^ |
|  | PC3 | −0.0014 | 0.006 | −0.256 | 7.98 × 10^−1^ |
|  | PC4 | −0.0065 | 0.006 | −1.18 | 2.38 × 10^−1^ |
|  | PC5 | −0.0010 | 0.006 | −0.18 | 8.55 × 10^−1^ |
|  | PC6 | −0.00018 | 0.006 | −0.032 | 9.74 × 10^−1^ |

**Supplementary Table S9.** Predictive value of PRS-Sz and Cannabis on CAPE-42 subscales, in Utrecht cohort. This table shows results for the linear regressions of various schizophrenia polygenic risk score on CAPE-42 subscales. We include the first 6 PC and sex as covariates for all analyses and age is included for all analyses of the CannabisQuest cohort. β= Beta, std.error = standard error, p = p value. PRS-Sz = polygenic risk score for schizophrenia. pt=p-value threshold.

.

Supplementary Table S10. Full mediation analysis of CAPE-42 subscales.

| **Dependent Variable** |  | **Independent Variable** | **label** | β | **se** | **z** | **pvalue** | **ci.lower** | **ci.upper** |
| --- | --- | --- | --- | --- | --- | --- | --- | --- | --- |
| **CAPEdep** | ~ | PRS | c1 | 0.0180 | 0.0070 | 2.5842 | 0.0098 | 0.0043 | 0.0316 |
| **CAPEdep** | ~ | Cannabis | b1 | 0.0101 | 0.0045 | 2.2550 | 0.0241 | 0.0013 | 0.0189 |
| **CAPEdep** | ~ | Sex |  | 0.1094 | 0.0140 | 7.8385 | 0.0000 | 0.0821 | 0.1368 |
| **CAPEdep** | ~ | PC1 |  | 0.0051 | 0.0099 | 0.5226 | 0.6013 | -0.0142 | 0.0245 |
| **CAPEdep** | ~ | PC2 |  | -0.0026 | 0.0071 | -0.3632 | 0.7164 | -0.0166 | 0.0114 |
| **CAPEdep** | ~ | PC3 |  | -0.0055 | 0.0094 | -0.5891 | 0.5558 | -0.0239 | 0.0128 |
| **CAPEdep** | ~ | PC4 |  | -0.0088 | 0.0093 | -0.9513 | 0.3414 | -0.0270 | 0.0093 |
| **CAPEdep** | ~ | PC5 |  | -0.0070 | 0.0072 | -0.9795 | 0.3273 | -0.0210 | 0.0070 |
| **CAPEdep** | ~ | PC6 |  | -0.0091 | 0.0127 | -0.7198 | 0.4716 | -0.0339 | 0.0157 |
| **CAPEneg** | ~ | PRS | c2 | 0.0148 | 0.0071 | 2.0956 | 0.0361 | 0.0010 | 0.0286 |
| **CAPEneg** | ~ | Cannabis | b2 | 0.0132 | 0.0045 | 2.9300 | 0.0034 | 0.0044 | 0.0220 |
| **CAPEneg** | ~ | Sex |  | 0.0249 | 0.0142 | 1.7549 | 0.0793 | -0.0029 | 0.0526 |
| **CAPEneg** | ~ | PC1 |  | 0.0122 | 0.0100 | 1.2152 | 0.2243 | -0.0074 | 0.0318 |
| **CAPEneg** | ~ | PC2 |  | -0.0034 | 0.0072 | -0.4635 | 0.6430 | -0.0176 | 0.0108 |
| **CAPEneg** | ~ | PC3 |  | -0.0029 | 0.0095 | -0.3079 | 0.7581 | -0.0216 | 0.0157 |
| **CAPEneg** | ~ | PC4 |  | -0.0161 | 0.0094 | -1.7141 | 0.0865 | -0.0346 | 0.0023 |
| **CAPEneg** | ~ | PC5 |  | -0.0051 | 0.0073 | -0.7081 | 0.4789 | -0.0194 | 0.0091 |
| **CAPEneg** | ~ | PC6 |  | -0.0078 | 0.0129 | -0.6068 | 0.5440 | -0.0330 | 0.0174 |
| **CAPEpos** | ~ | PRS | c3 | 0.0108 | 0.0046 | 2.3135 | 0.0207 | 0.0016 | 0.0199 |
| **CAPEpos** | ~ | Cannabis | b3 | 0.0040 | 0.0030 | 1.3578 | 0.1745 | -0.0018 | 0.0099 |
| **CAPEpos** | ~ | Sex |  | -0.0006 | 0.0093 | -0.0643 | 0.9487 | -0.0189 | 0.0177 |
| **CAPEpos** | ~ | PC1 |  | 0.0002 | 0.0066 | 0.0270 | 0.9785 | -0.0127 | 0.0131 |
| **CAPEpos** | ~ | PC2 |  | -0.0027 | 0.0048 | -0.5675 | 0.5704 | -0.0120 | 0.0066 |
| **CAPEpos** | ~ | PC3 |  | -0.0183 | 0.0063 | -2.9290 | 0.0034 | -0.0306 | -0.0061 |
| **CAPEpos** | ~ | PC4 |  | 0.0017 | 0.0062 | 0.2777 | 0.7812 | -0.0104 | 0.0138 |
| **CAPEpos** | ~ | PC5 |  | 0.0038 | 0.0048 | 0.8046 | 0.4211 | -0.0055 | 0.0132 |
| **CAPEpos** | ~ | PC6 |  | 0.0271 | 0.0085 | 3.2032 | 0.0014 | 0.0105 | 0.0437 |
| **Cannabis** | ~ | PRS | a | 0.0969 | 0.0437 | 2.2196 | 0.0264 | 0.0113 | 0.1825 |
| **Cannabis** | ~ | Sex |  | -0.4086 | 0.0876 | -4.6661 | 0.0000 | -0.5803 | -0.2370 |
| **Cannabis** | ~ | PC1 |  | 0.0379 | 0.0624 | 0.6069 | 0.5439 | -0.0845 | 0.1603 |
| **Cannabis** | ~ | PC2 |  | -0.0205 | 0.0451 | -0.4548 | 0.6493 | -0.1089 | 0.0679 |
| **Cannabis** | ~ | PC3 |  | 0.0151 | 0.0600 | 0.2520 | 0.8011 | -0.1025 | 0.1327 |
| **Cannabis** | ~ | PC4 |  | 0.1171 | 0.0588 | 1.9906 | 0.0465 | 0.0018 | 0.2324 |
| **Cannabis** | ~ | PC5 |  | -0.0218 | 0.0448 | -0.4878 | 0.6257 | -0.1096 | 0.0659 |
| **Cannabis** | ~ | PC6 |  | -0.2006 | 0.0823 | -2.4357 | 0.0149 | -0.3619 | -0.0392 |
| **CAPEdep** | ~~ | CAPEdep |  | 0.0550 | 0.0023 | 24.0292 | 0.0000 | 0.0505 | 0.0595 |
| **CAPEneg** | ~~ | CAPEneg |  | 0.0566 | 0.0024 | 24.0252 | 0.0000 | 0.0520 | 0.0612 |
| **CAPEpos** | ~~ | CAPEpos |  | 0.0245 | 0.0010 | 24.0377 | 0.0000 | 0.0225 | 0.0265 |
| **Cannabis** | ~~ | Cannabis |  | 2.6862 | 0.1013 | 26.5236 | 0.0000 | 2.4877 | 2.8847 |
| **CAPEdep** | ~~ | CAPEneg |  | 0.0382 | 0.0020 | 19.1766 | 0.0000 | 0.0343 | 0.0420 |
| **CAPEdep** | ~~ | CAPEpos |  | 0.0201 | 0.0012 | 16.3308 | 0.0000 | 0.0177 | 0.0225 |
| **CAPEneg** | ~~ | CAPEpos |  | 0.0197 | 0.0012 | 15.8555 | 0.0000 | 0.0172 | 0.0221 |
| **PRS** | ~~ | PRS |  | 0.9994 | 0.0000 | NA | NA | 0.9994 | 0.9994 |
| **PRS** | ~~ | Sex |  | 0.0107 | 0.0000 | NA | NA | 0.0107 | 0.0107 |
| **PRS** | ~~ | PC1 |  | -0.0523 | 0.0000 | NA | NA | -0.0523 | -0.0523 |
| **PRS** | ~~ | PC2 |  | 0.0346 | 0.0000 | NA | NA | 0.0346 | 0.0346 |
| **PRS** | ~~ | PC3 |  | -0.0319 | 0.0000 | NA | NA | -0.0319 | -0.0319 |
| **PRS** | ~~ | PC4 |  | -0.0066 | 0.0000 | NA | NA | -0.0066 | -0.0066 |
| **PRS** | ~~ | PC5 |  | -0.0044 | 0.0000 | NA | NA | -0.0044 | -0.0044 |
| **PRS** | ~~ | PC6 |  | -0.0023 | 0.0000 | NA | NA | -0.0023 | -0.0023 |
| **Sex** | ~~ | Sex |  | 0.2500 | 0.0000 | NA | NA | 0.2500 | 0.2500 |
| **Sex** | ~~ | PC1 |  | -0.0132 | 0.0000 | NA | NA | -0.0132 | -0.0132 |
| **Sex** | ~~ | PC2 |  | -0.0066 | 0.0000 | NA | NA | -0.0066 | -0.0066 |
| **Sex** | ~~ | PC3 |  | -0.0123 | 0.0000 | NA | NA | -0.0123 | -0.0123 |
| **Sex** | ~~ | PC4 |  | 0.0039 | 0.0000 | NA | NA | 0.0039 | 0.0039 |
| **Sex** | ~~ | PC5 |  | 0.0133 | 0.0000 | NA | NA | 0.0133 | 0.0133 |
| **Sex** | ~~ | PC6 |  | -0.0035 | 0.0000 | NA | NA | -0.0035 | -0.0035 |
| **PC1** | ~~ | PC1 |  | 0.9994 | 0.0000 | NA | NA | 0.9994 | 0.9994 |
| **PC1** | ~~ | PC2 |  | -0.0825 | 0.0000 | NA | NA | -0.0825 | -0.0825 |
| **PC1** | ~~ | PC3 |  | 0.5587 | 0.0000 | NA | NA | 0.5587 | 0.5587 |
| **PC1** | ~~ | PC4 |  | -0.4443 | 0.0000 | NA | NA | -0.4443 | -0.4443 |
| **PC1** | ~~ | PC5 |  | 0.1180 | 0.0000 | NA | NA | 0.1180 | 0.1180 |
| **PC1** | ~~ | PC6 |  | 0.6706 | 0.0000 | NA | NA | 0.6706 | 0.6706 |
| **PC2** | ~~ | PC2 |  | 0.9994 | 0.0000 | NA | NA | 0.9994 | 0.9994 |
| **PC2** | ~~ | PC3 |  | -0.0920 | 0.0000 | NA | NA | -0.0920 | -0.0920 |
| **PC2** | ~~ | PC4 |  | -0.1418 | 0.0000 | NA | NA | -0.1418 | -0.1418 |
| **PC2** | ~~ | PC5 |  | 0.0366 | 0.0000 | NA | NA | 0.0366 | 0.0366 |
| **PC2** | ~~ | PC6 |  | 0.0813 | 0.0000 | NA | NA | 0.0813 | 0.0813 |
| **PC3** | ~~ | PC3 |  | 0.9994 | 0.0000 | NA | NA | 0.9994 | 0.9994 |
| **PC3** | ~~ | PC4 |  | -0.3726 | 0.0000 | NA | NA | -0.3726 | -0.3726 |
| **PC3** | ~~ | PC5 |  | 0.0475 | 0.0000 | NA | NA | 0.0475 | 0.0475 |
| **PC3** | ~~ | PC6 |  | 0.6386 | 0.0000 | NA | NA | 0.6386 | 0.6386 |
| **PC4** | ~~ | PC4 |  | 0.9994 | 0.0000 | NA | NA | 0.9994 | 0.9994 |
| **PC4** | ~~ | PC5 |  | -0.1095 | 0.0000 | NA | NA | -0.1095 | -0.1095 |
| **PC4** | ~~ | PC6 |  | -0.6702 | 0.0000 | NA | NA | -0.6702 | -0.6702 |
| **PC5** | ~~ | PC5 |  | 0.9994 | 0.0000 | NA | NA | 0.9994 | 0.9994 |
| **PC5** | ~~ | PC6 |  | 0.2059 | 0.0000 | NA | NA | 0.2059 | 0.2059 |
| **PC6** | ~~ | PC6 |  | 0.9994 | 0.0000 | NA | NA | 0.9994 | 0.9994 |
| **CAPEdep** | ~1 |  |  | 2.4021 | 0.0232 | 103.6698 | 0.0000 | 2.3567 | 2.4476 |
| **CAPEneg** | ~1 |  |  | 3.0253 | 0.0235 | 128.7388 | 0.0000 | 2.9792 | 3.0714 |
| **CAPEpos** | ~1 |  |  | 3.1970 | 0.0155 | 206.6699 | 0.0000 | 3.1667 | 3.2273 |
| **Cannabis** | ~1 |  |  | 1.4561 | 0.1396 | 10.4323 | 0.0000 | 1.1825 | 1.7297 |
| **PRS** | ~1 |  |  | 0.0000 | 0.0000 | NA | NA | 0.0000 | 0.0000 |
| **Sex** | ~1 |  |  | 1.5057 | 0.0000 | NA | NA | 1.5057 | 1.5057 |
| **PC1** | ~1 |  |  | 0.0000 | 0.0000 | NA | NA | 0.0000 | 0.0000 |
| **PC2** | ~1 |  |  | 0.0000 | 0.0000 | NA | NA | 0.0000 | 0.0000 |
| **PC3** | ~1 |  |  | 0.0000 | 0.0000 | NA | NA | 0.0000 | 0.0000 |
| **PC4** | ~1 |  |  | 0.0000 | 0.0000 | NA | NA | 0.0000 | 0.0000 |
| **PC5** | ~1 |  |  | 0.0000 | 0.0000 | NA | NA | 0.0000 | 0.0000 |
| **PC6** | ~1 |  |  | 0.0000 | 0.0000 | NA | NA | 0.0000 | 0.0000 |
| **Cannabis_IDE_dep** | := | a*b1 | Cannabis_IDE_dep | 0.0010 | 0.0006 | 1.5836 | 0.1133 | -0.0002 | 0.0022 |
| **Cannabis_IDE_neg** | := | a*b2 | Cannabis_IDE_neg | 0.0013 | 0.0007 | 1.7685 | 0.0770 | -0.0001 | 0.0027 |
| **Cannabis_IDE_pos** | := | a*b3 | Cannabis_IDE_pos | 0.0004 | 0.0003 | 1.1585 | 0.2467 | -0.0003 | 0.0011 |
| **sum_IDE_Cannabis** | := | (a*b1)+(a*b2)+(a*b3) | sum_IDE_Cannabis | 0.0027 | 0.0016 | 1.7052 | 0.0882 | -0.0004 | 0.0057 |
| **total** | := | (c1+(a*b1))+(c2+(a*b2))+(c3+(a*b3)) | total | 0.0462 | 0.0160 | 2.8787 | 0.0040 | 0.0147 | 0.0776 |

**Supplementary Table S10.** Full mediation analysis of CAPE-42 subscales. We include the first 6 PC and Sex as covariates for all analyses and age is included for all analyses of the Utrecht cohort.

Supplementary Table S11. Full results of sensitivity analysis.

| IMAGEN |  | β | std.error | statistic | p |
| --- | --- | --- | --- | --- | --- |
| Model 1 |  |  |  |  |  |
|  | PRS-Sz | 0.014603 | 0.005248 | 2.782261 | ***5.49 × 10^−3^*** |
|  | PRS-Can | 0.006256 | 0.005268 | 1.187526 | 2.35 × 10^−1^ |
|  | SEX | 0.030118 | 0.010454 | 2.880897 | ***4.04 × 10^−3^*** |
|  | PC1 | 0.005636 | 0.007433 | 0.758275 | 4.48 × 10^−1^ |
|  | PC2 | −0.003781 | 0.005382 | −0.702496 | 4.83 × 10^−1^ |
|  | PC3 | −0.010362 | 0.007069 | −1.465861 | 1.43 × 10^−1^ |
|  | PC4 | −0.006082 | 0.006998 | −0.869097 | 3.85 × 10^−1^ |
|  | PC5 | −0.002224 | 0.005391 | −0.412430 | 6.80 × 10^−1^ |
|  | PC6 | 0.005439 | 0.009558 | 0.569034 | 5.69 × 10^−1^ |
| Model 2 |  |  |  |  |  |
|  | PRS-Sz | 0.094862 | 0.043809 | 2.165354 | ***3.05 × 10^−2^*** |
|  | PRS-Can | 0.069959 | 0.043575 | 1.605476 | 1.09 × 10^−1^ |
|  | SEX | −0.406206 | 0.087838 | −4.624470 | ***4.10 × 10^−6^*** |
|  | PC1 | 0.037177 | 0.062608 | 0.593801 | 5.53 × 10^−1^ |
|  | PC2 | −0.021686 | 0.045232 | −0.479433 | 6.32 × 10^−1^ |
|  | PC3 | 0.01423 | 0.060177 | 0.236464 | 8.13 × 10^−1^ |
|  | PC4 | 0.121357 | 0.059062 | 2.05474 | ***4.01 × 10^−2^*** |
|  | PC5 | −0.019007 | 0.044949 | −0.422855 | 6.72 × 10^−1^ |
|  | PC6 | −0.186235 | 0.082801 | −2.249182 | ***2.47 × 10^−2^*** |
| Model 3 |  |  |  |  |  |
|  | PRS-Sz | 0.01385 | 0.005463 | 2.535454 | ***1.14 × 10^−2^*** |
|  | PRS-Can | 0.004674 | 0.005454 | 0.857 | 3.92 × 10^−1^ |
|  | Cannabis use | 0.008955 | 0.003355 | 2.669299 | ***7.72 × 10^−3^*** |
|  | SEX | 0.035686 | 0.010955 | 3.257683 | ***1.16 × 10^−3^*** |
|  | PC1 | 0.005897 | 0.007745 | 0.761414 | 4.47 × 10^−1^ |
|  | PC2 | −0.002998 | 0.005552 | −0.540051 | 5.89 × 10^−1^ |
|  | PC3 | −0.009627 | 0.007392 | −1.302329 | 1.93 × 10^−1^ |
|  | PC4 | −0.006330 | 0.007245 | −0.873767 | 3.82 × 10^−1^ |
|  | PC5 | −0.001636 | 0.005573 | −0.293511 | 7.69 × 10^−1^ |
|  | PC6 | 0.010135 | 0.010071 | 1.006395 | 3.14 × 10^−1^ |

**Supplementary Table S11**. Full sensitivity analysis. Here we include the PRS-Can as a potential confounder in the three linear models (model 1-2-3), in the IMAGEN cohort. As in other analyses, we include the first 6 PC and Sex as covariates for all analyses and age is included for all analyses of the Utrecht cohort.
