# Supplementary figures and images for: Independent contribution of polygenic risk for schizophrenia and cannabis use in predicting psychotic-like experiences in young adulthood: testing gene × environment moderation and mediation
Source: Psychol Med. 2021 Sep 23;53(5):1759–69. doi: 10.1017/S0033291721003378 (PMC10106286; doi:10.1017/S0033291721003378)

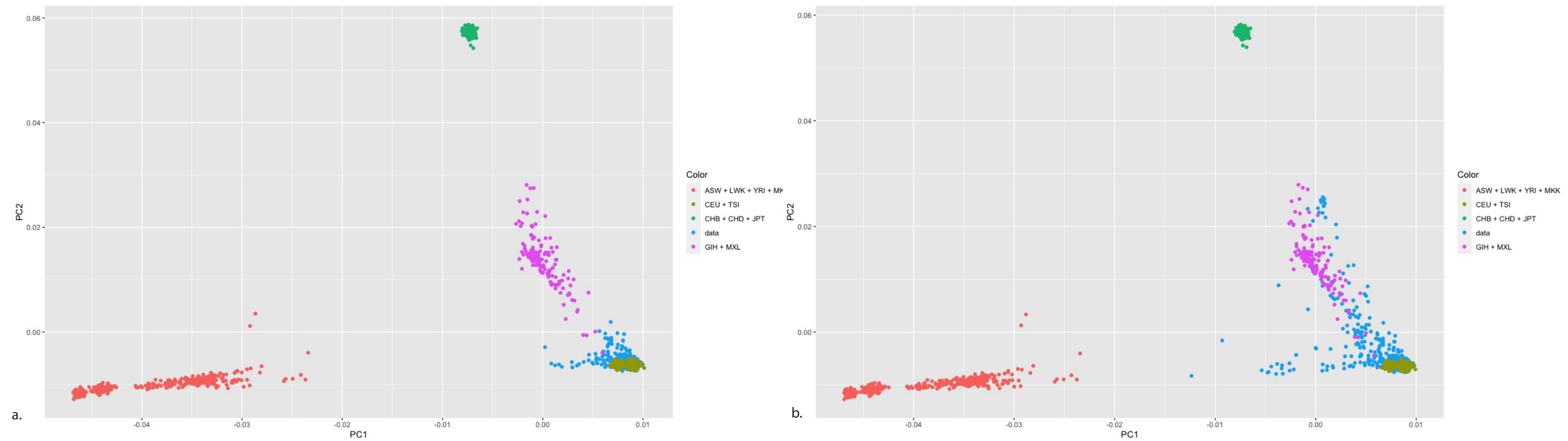

Supplement: Supplementary file 1 [file S0033291721003378sup.zip › S0033291721003378sup001.pdf]
